# Transient tissue residency and lymphatic egress define human CD56^bright^ NK cell homeostasis

**DOI:** 10.1038/s41590-025-02290-9

**Published:** 2025-10-14

**Authors:** Annika Niehrs, Laura Hertwig, Marcus Buggert, Isabella Nordström, Maura Statzu, M. Betina Pampena, Sadia Samer, James J. Knox, Benedikt Strunz, Dan Sun, Son Nguyen, Claudia Janoschka, Yafei Xing, Vincent H. Wu, Ernesto Sparrelid, Arlisa Alisjahbana, Yu Gao, Natalie Sleiers, Otto Strauss, Iva Filipovic, Andrea Ponzetta, Itzel Medina-Andrade, Vera Nilsén, Carl Jorns, Martin Cornillet, Christopher Maucourant, Christoph Ziegenhain, Julia Hengst, George Tweet, Kyle Kroll, Gregory J. Golden, Heiner Wedemeyer, Murat Kürtüncü, Yoav Dori, Maxim G. Itkin, Luisa Klotz, Marie Schaffer, Bo-Göran Ericzon, Martin A. Ivarsson, Mirko Paiardini, Greg Nowak, Tim Willinger, R. Keith Reeves, Michael R. Betts, Niklas K. Björkström

**Affiliations:** 1 Center for Infectious Medicine, Department of Medicine Huddinge, Karolinska Institutet, Karolinska University Hospital, Stockholm, Sweden; 2 Department of Microbiology, Perelman School of Medicine, University of Pennsylvania, Philadelphia, PA USA; 3 Division of Microbiology and Immunology, Emory National Primate Research Center, Emory University, Atlanta, GA USA; 4 Institute for Immunology, University of Pennsylvania, Perelman School of Medicine, Philadelphia, PA USA; 5 Department of Pathology, Perelman School of Medicine, University of Pennsylvania, Philadelphia, PA USA; 6 Department of Neurology with Institute of Translational Neurology, University Hospital Münster, Muenster, Germany; 7 Department of Clinical Science, Intervention and Technology, Division of Surgery, Karolinska Institutet, Karolinska University Hospital, Stockholm, Sweden; 8 Department of Transplantation, Karolinska University Hospital, Stockholm, Sweden; 9 Department of Medical Biochemistry and Biophysics, Karolinska Institutet, Stockholm, Sweden; 10 Department of Gastroenterology, Hepatology and Endocrinology, Hannover Medical School, Hannover, Germany; 11 Center for Virology and Vaccine Research, Beth Israel Deaconess Medical Center, Harvard Medical School, Boston, MA USA; 12 Division of Innate and Comparative Immunology, Center for Human Systems Immunology, Department of Surgery, Duke University School of Medicine, Durham, NC USA; 13 Department of Gastroenterology and Hepatology, Essen University Hospital, Essen, Germany; 14 Department of Neurology, Istanbul Faculty of Medicine, Istanbul University, Istanbul, Turkey; 15 Division of Cardiology, Department of Pediatrics, The Children’s Hospital of Philadelphia, Philadelphia, PA USA; 16 Department of Radiology, Hospital of the University of Pennsylvania, Philadelphia, PA USA; 17 Division of Transplantation Surgery, Department of Clinical Science, Intervention and Technology (CLINTEC), Karolinska Institutet, Stockholm, Sweden; 18 Department of Pathology and Laboratory Medicine, Emory University, Atlanta, GA USA; 19 Columbia Center for Translational Immunology (CCTI), Columbia University, New York, NY USA; 20 Ragon Institute of Massachusetts General Hospital, MIT and Harvard, Cambridge, MA USA; 21 Department of Surgery, Duke University School of Medicine, Durham, NC USA; 22 Present Address: Department of Genomic Medicine Unit, Sanofi, Waltham, MA USA; 23 Present Address: Division of Life Science, Hong Kong University of Science and Technology, Hong Kong, China; 24 Present Address: Department of Biology and Program in Biochemistry, Bowdoin College, Brunswick, ME USA

**Keywords:** Lymphoid tissues, NK cells

## Abstract

Human tissue-resident (TR) CD56^bright^ natural killer (NK) cells can be identified by expression of integrins and chemokine receptors inferred from murine studies, but many aspects of their homeostasis are unclear. Here we used an integrated approach of dynamic human, humanized mouse and non-human primate models and sampling of efferent lymph fluid to determine recirculation and TR patterns of human NK cells. By intravascular labeling, we showed that CD56^bright^ NK cells access tissue niches at steady state. Furthermore, in human liver transplantation, donor-derived CD56^bright^ NK cells represent the dominant TR NK cell population early after transplantation, but are replaced over time by infiltrating recipient NK cells that establish TR traits, a process partly regulated by Runx3. Transient TR CD56^bright^ NK cells recirculated via lymphatics, displaying a consistent phenotype detectable in draining lymph nodes and efferent lymph fluid, and waned from peripheral blood on lymph node egress blockade. Finally, CD56^dim^ NK cells, constrained to vasculature at steady state, entered lymph nodes upon inflammation. This study provides a mechanistic framework for the transient tissue residency and recirculation pattern of human NK cell populations.

## Abstract (web summary)

Here the authors map the dynamics of human NK cell residency and recirculation, showing that CD56^bright^ NK cells transiently occupy tissues and recirculate via lymphatics, whereas CD56^dim^ NK cells remain vascular except during inflammation.

## Main

Substantial fractions of both adaptive and innate lymphocytes permanently reside in peripheral organs at steady state, where they function as an early defense against infections and cancer, as well as contributing to homeostasis^1,2^. Human natural killer (NK) cells represent one group of innate immune cells that is present across multiple human tissues^1,3^. NK cells can be subdivided into CD56^bright^ and CD56^dim^ NK cell subsets possessing distinct phenotypic and functional properties. CD56^dim^ NK cells are efficient cytotoxic effector cells due to their high expression of cytolytic granule proteins, such as perforin and granzyme B, whereas CD56^bright^ NK cells contribute to homeostatic and immunomodulatory processes through secretion of cytokines^4^.

Parabiosis experiments in mice have revealed the existence of tissue-resident (TR) NK cells^5,6^ and corresponding subsets of cells have been phenotypically identified in human lymphoid and peripheral tissues^7–14^. These studies suggest that each organ contains unique types of putative TR NK cells that function in local microenvironments and display properties more reminiscent of CD56^bright^ than of CD56^dim^ NK cells^3,15^. This challenges the classic view of NK cells as continuously circulating through the body, performing immunosurveillance. However, it remains poorly understood whether CD56^bright^ and CD56^dim^ NK cells display different extravasation capacities or recirculation patterns at steady state, potentially resulting in the high abundance of CD56^bright^ NK cells in tissue. In addition, the longevity of human NK cell tissue residency is not understood. Murine studies suggest that NK cell tissue residency is long term in certain organs but more transient in others^5^. Similarly, murine T cell tissue residency can also be both long term and more transient, the latter in particular being the case for regulatory T cells^16^. Furthermore, it remains elusive whether TR NK cells re-enter the circulation and whether this is facilitated via lymph fluid rather than through the vascular network.

To address these questions, we first mapped human NK cells across multiple tissues, subsequently investigated the dynamics and longevity of NK cell tissue residency and finally assessed recirculation and tissue access patterns of the cells using a combination of human, macaque and humanized mouse models. We showed that CD56^bright^ but not CD56^dim^ NK cells access the tissue parenchyma and reside in the extravascular space within organs during steady-state conditions. Human liver CD56^bright^ NK cells were transiently TR and replaced with newly recruited cells from the circulation that adapted TR traits over time. Finally, although conventional CD56^dim^ NK cells were confined to the vasculature, liver CD56^bright^ NK cells recirculated via lymph fluid, displaying a consistent liver tissue imprint that was observed within draining lymph nodes and efferent lymph fluid. Together, these results identify distinct tissue residency and recirculation behaviors of human NK cell subpopulations.

## Results

### CD56^bright^ NK cells are abundant in peripheral tissues

**Fig. 1:**
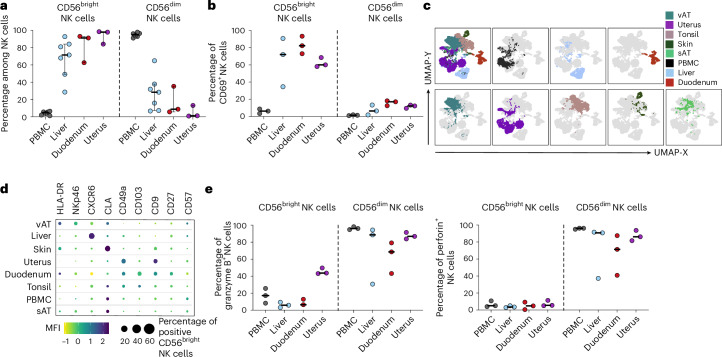
CD56^bright^ NK cells from distinct human tissues have common and unique TR molecule expression. **a**, Percentage of CD56^bright^ (left) and CD56^dim^ (right) NK cells out of total NK cells in indicated human tissues. Each dot represents one individual donor (*n* = 3 for duodenum and uterus; *n* = 7 for PBMCs and liver). The line indicates the median and the bars represent the interquartile range (IQR). **b**, Frequency of CD69^+^ NK cells within NK cell subsets and tissues: CD56^bright^ (left) and CD56^dim^ (right) NK cells. Each dot represents one individual donor (*n* = 3) and the line indicates the median. **c**, CD56^bright^ NK cells from various organs of 3–6 donors were downsampled to 300–500 events per individual, concatenated (1,000–3,000 events in total per organ) and visualized by dimensionality reduction using Uniform Manifold Approximation and Projection (UMAP). **d**, Balloon plot showing expression of nine surface receptors on CD56^bright^ NK cells across human tissues (*n* = 3–6). The color scale represents the row *z*-score of median fluorescence intensity and the dot size equals the proportion of receptor-positive CD56^bright^ NK cells. **e**, Left: frequency of expression of granzyme B within NK cell subsets and tissues: CD56^bright^ (left) and CD56^dim^ (right). Right: frequency of expression of perforin within NK cell subsets and tissues: CD56^bright^ (left) and CD56^dim^ (right). Each dot represents one individual donor (*n* = 3). The line represents the median. CLA, cutaneous lymphocyte-associated antigen; vAT, visceral adipose tissue; sAT, subcutaneous adipose tissue.

Human innate immune cells, including NK cells, circulate in peripheral blood (PB) but are also highly abundant in many peripheral tissues. Phenotypes of NK cells within the circulation and peripheral tissues have been described as differing substantially, with CD56^dim^ NK cells dominating in PB and CD56^bright^ NK cells expressing TR markers being enriched within human tissues^3,5–9,12,14,17–20^. As a point of departure toward mapping tissue accessibility and dynamics of recirculation routes, we first corroborated published work by characterizing NK cell subset composition and phenotypes across different human tissues, as well as in PB, using high-dimensional flow cytometry. Human NK cells were defined as CD45^+^CD14^−^CD15^−^CD19^−^CD3^−^CD56^+^ cells and distinguished from other non-NK innate lymphoid cells (ILCs) using CD94 and CD127 (Supplementary Fig. 1a). Subsequently, NK cells were divided into CD56^bright^ and CD56^dim^ NK cell subsets and the expression of TR markers was assessed for each subset (Supplementary Fig. 1b). In accordance with previous studies^3,6,8,19,20^, we observed a high abundance of CD56^dim^ NK cells in PB, whereas CD56^bright^ NK cells were broadly prevalent in peripheral tissues (Fig. 1a). In addition, CD56^bright^ NK cells expressed TR markers, which were unique to the individual organs, apart from CD69 which was expressed in CD56^bright^ NK cells across all organs (Fig. 1b–d). Specifically, CD56^bright^ NK cells expressed cutaneous lymphocyte-associated antigen in skin and subcutaneous adipose tissue (sAT), CXCR6 in liver, NKp46 in visceral adipose tissue (vAT), CD9 and CD49a in the uterus, CD103 and CD49a in the tonsils and CD27, CD49a, CD103 and human leukocyte antigen (HLA)-DR in the duodenum (Fig. 1d and Supplementary Fig. 1c). In contrast, only a few CD56^dim^ NK cells displayed expression of tissue-residency markers like CD69 (Fig. 1b). The CD56^dim^ NK cell compartment was characterized by the expression of the cytotoxic effector molecules granzyme B and perforin, which exhibited lower expression levels or were completely absent within CD56^bright^ NK cells (Fig. 1e).

In summary, CD56^bright^ NK cells are the dominant NK cell type across multiple human tissues and possess a TR phenotype that reflects the unique tissue microenvironment.

### CD56^bright^ NK cells reside in the tissue parenchyma during steady state

**Fig. 2:**
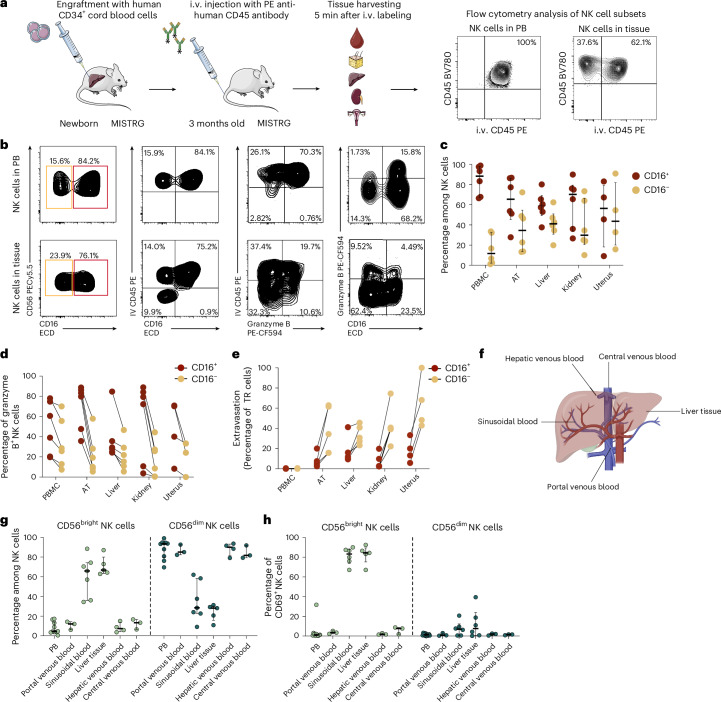
CD56^bright^ NK cells can access the tissue parenchyma in steady-state conditions. **a**, Schema of experimental workflow for MISTRG humanized mouse experiments and representative flow cytometry plots of i.v. labeling. **b**, Representative flow cytometry plots showing CD56^+^CD16^+^ (red) and CD56^+^CD16^−^ (orange) NK cells, i.v. CD45-labeled NK cells versus CD16 expression, i.v. CD45-labeled cells versus granzyme B expression and granzyme B-expressing CD16^+^ and CD16^−^ NK cells (left to right) in PB and example tissues. **c**,**d**, Summary data for NK cell subsets from indicated tissues (**c**) and their expression of granzyme B (**d**). Each dot represents an individual mouse (*n* = 4 for uterus; *n* = 6 for PBMCs, AT, liver and kidney). The line in **c** indicates the median and the error bars display the IQR. The lines in **d** connect matched values from the same mouse. **e**, Summary data for frequency of extravasated cells within NK cell subsets, shown as proportion of non-i.v. CD45-labeled NK cells. Each dot represents an individual mouse (*n* = 4 for uterus; *n* = 6 for PBMCs, AT, liver and kidney). The lines connect CD16^+^ and CD16^−^ NK cell values from the same mouse. **f**, Sample collection scheme for afferent (portal venous) and efferent (hepatic venous and central venous) blood of the human liver and liver tissue. **g**,**h**, Summary data for NK cell subset distribution (**g**) and expression of CD69 within NK cell subsets (**h**) from indicated sources: CD56^bright^ (left) and CD56^dim^ (right) NK cells. Each dot represents one individual donor (*n* = 3 for portal venous blood, hepatic venous blood and central venous blood; *n* = 5 for liver tissue and sinusoidal blood; *n* = 9 for PBMCs). The line indicates the median and the bars depict the IQR. Illustrations in **a** from Servier Medical Art (https://smart.servier.com/); panel **a** and **f** created using BioRender.com.

As there was a clear dominance of CD56^bright^ NK cells within human tissues, we next wanted to determine whether this NK cell subset had better access to tissues than CD56^dim^ NK cells at steady state. To experimentally determine tissue access of different NK cell subsets in vivo, we used the MISTRG humanized mouse model expressing human cytokines through gene knock-in^21–24^. Newborn MISTRG mice, which have previously been shown to support robust human NK cell development^22,23^, were engrafted with human CD34^+^ hematopoietic stem and progenitor cells (Fig. 2a). Then 3 months after engraftment, we performed intravascular (i.v.) labeling of human immune cells in vivo using a phycoerythrin (PE)-conjugated, anti-human CD45 antibody^25^. Analysis of 3-month-old mice revealed that NK cells were present in PB as well as in tissue (Fig. 2b,c). As expected, all NK cells in PB were stained with the anti-human CD45-PE antibody after i.v. administration (Fig. 2b). In contrast, a subset of human NK cells isolated from tissue was protected from antibody labeling, consistent with being located in the extravascular space (Fig. 2b). In line with the NK cell subset composition in humans, human CD56^+^CD16^−^ NK cells (CD56^bright^ like) isolated from MISTRG mice were more abundant in tissues compared to PB, whereas CD56^+^CD16^+^ NK cells (CD56^dim^ like) were more prevalent in PB and expressed higher levels of granzyme B than CD56^+^CD16^−^ NK cells (Fig. 2c,d). Interestingly, the main NK cell subset that remained unlabeled by the i.v. CD45-PE antibody was the CD56^+^CD16^−^ subset, indicating that this subset is localized within the extravascular tissue parenchyma (Fig. 2e).

To determine how NK cells enter and exit the tissue, we assessed the NK cell composition of portal venous blood (afferent blood of the liver), sinusoidal blood, hepatic venous blood (efferent venous blood of the liver) and central venous blood, and compared it to the NK cell composition within the liver tissue (Fig. 2f,g). CD56^dim^ NK cells were present at similar frequencies in portal, hepatic and central venous blood, indicating that these cells have overall limited tissue access and enter and leave organs via the circulation. In contrast, CD56^bright^ NK cells represented most of the NK cells within liver tissue and sinusoidal blood, but were less abundant within PB as well as portal, hepatic and central venous blood (Fig. 2g). These data indicate that CD56^bright^ NK cells do not primarily recirculate via the vasculature network, but use a distinct route of trafficking. Furthermore, CD56^bright^ NK cells within the sinusoidal blood and liver tissue displayed high expression levels of CD69, indicative of a tissue residency phenotype (Fig. 2h).

Thus, CD56^bright^ NK cells reside in the tissue parenchyma at steady state consistent with the exit from the local vasculature and adaptation of a TR expression profile, whereas CD56^dim^ NK cells remain in the vasculature.

### Donor-derived cells are the major liver immune population after transplantation

**Fig. 3:**
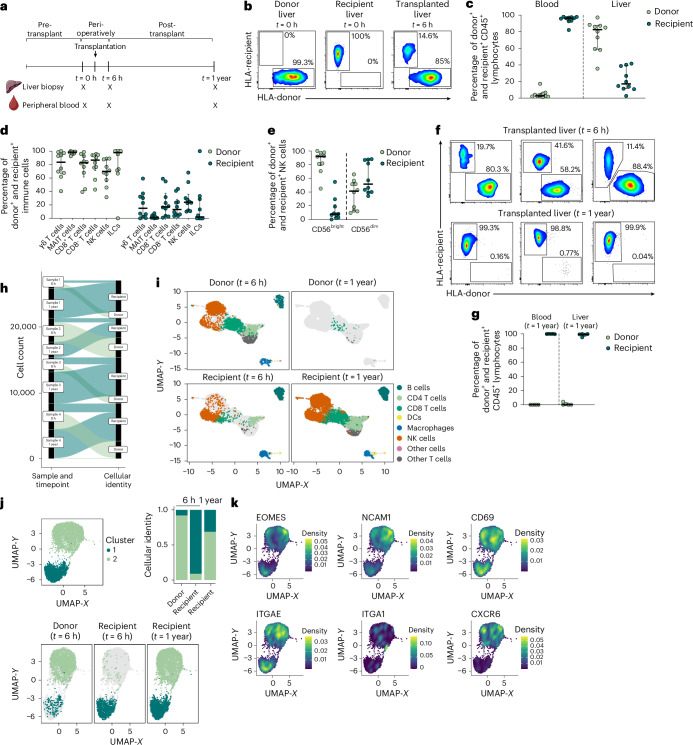
Infiltrating NK cells replace existing TR cells over time. **a**, Schematic overview of sample collection. Liver tissue and PB were collected before liver transplantation as well as shortly after (*t* = 6 h) and 1 year after transplantation. **b**, Representative flow cytometry plot of HLA-donor^+^ and HLA-recipient^+^ CD45^+^ lymphocytes at *t* = 0 h and *t* = 6 h. Left: donor liver, *t* = 0 h. Middle: recipient liver, *t* = 0 h. Right: transplanted liver, *t* = 6 h. **c**,**d**, Percentage of HLA-donor^+^ (light green) and HLA-recipient^+^ (dark green) within overall CD45^+^ lymphocytes (**c**) in the blood (left) and liver (right) and specific immune cell subsets (**d**) in the liver. **e**, Percentage of HLA-donor^+^ (light green) and HLA-recipient^+^ (dark green) cells in CD56^bright^ and CD56^dim^ NK cells in the liver. Each dot represents one individual (*n* = 10). The line indicates the median and the error bars illustrate the IQR. **f**, Representative flow cytometry plots of HLA-donor or HLA-recipient expression in transplanted liver samples 6 h (top) and 1 year (bottom) after transplantation. **g**, Percentage of HLA-donor^+^ (light green) and HLA-recipient^+^ (dark green) cells of overall CD45^+^ lymphocytes at 1 year post-transplantation (*t* = 1 year) for blood (left) and liver (right). Each dot represents one individual donor (*n* = 5). The line indicates the median. ScRNA-seq of liver biposies (*n* = 4) was conducted at two timepoints after liver transplantation (*t* = 6 h and *t* =1 year). **h**, Donor and recipient identity of cells for each sample and timepoint determined using the Vireo package, leveraging cellular genotyping data based on the most common SNPs from the 1000 Genomes Project. **i**, UMAP of major immune populations identified at distinct timepoints (*t* = 6 h (left) and *t* *=* 1 year (rigt)) according to donor (top) and recipient (bottom) cell identity. **j**, Two clusters of NK cells (custer 1 = dark green; cluster 2 = light green) identified within transplanted samples. The cell identity panel for each cluster was assessed at different timepoints after transplantation. **k**, Differential expression of multiple TR markers assessed between the distinct NK cell clusters. Illustrations in **a** from Servier Medical Art (https://smart.servier.com/); panel **a** created using BioRender.com. MAIT, mucosal-associated invariant T cell.

After mapping CD56^bright^ NK cells across different human tissues, we next assessed the dynamics of TR CD56^bright^ NK cells by tracking their turnover within human tissues using a liver transplantation cohort. Liver transplantation can be carried out despite HLA mismatch, allowing us to track donor-derived and recipient-derived immune cells by staining for the respective HLA molecules (Supplementary Fig. 2a,b and Supplementary Table 1). PB and liver biopsies were obtained at three different timepoints: before transplantation (*t* = 0 h), shortly after transplantation (*t* = 6 h) and 1 year after transplantation (*t* = 1 year) (Fig. 3a). Before transplantation (*t* = 0 h), all immune cells within the donor and recipient liver expressed only the corresponding HLA-donor and HLA-recipient molecules (Fig. 3b). However, 6 h after transplantation, all cells across multiple immune subsets, including γσ T cells, CD8^+^ and CD8^−^ T cells, NK cells and ILCs, were of both donor and recipient origin, but with a higher percentage of HLA-donor^+^ immune cells in the liver (Fig. 3b–d). In contrast, and as expected, PB immune cells were almost exclusively of recipient origin 6 h after transplantation (Supplementary Fig. 2c). In PB, CD56^dim^ NK cells were primarily of recipient origin whereas the CD56^bright^ NK cell subset consisted of both donor-derived and recipient-derived cells (Supplementary Fig. 2c). In line with being TR, the origin of CD56^bright^ and CD56^dim^ NK cells was reversed in the liver compared to PB, with a dominance of donor-derived CD56^bright^ NK cells (Fig. 3e). In contrast to 6 h after transplantation, donor-derived lymphocytes were barely detectable within the liver 1 year after transplantation (Fig. 3f,g). The few remaining donor-derived lymphocytes were primarily T cells (Supplementary Fig. 2d), indicating the existence of long-term TR cells. Importantly, 1-year liver biopsies were carried out as routine procedures in all liver transplant recipients included and biopsies did not display signs of graft rejection.

In summary, liver CD56^bright^ NK cells represent transiently TR immune cells that are replenished from the circulation over time.

### Recipient cells infiltrating the liver adopt a TR transcriptome

To analyze NK cells within the liver in more detail and to determine how the recipient-infiltrating NK cells develop over time, we performed single-cell RNA sequencing (scRNA-seq) of total CD45^+^ cells from samples of four different transplanted livers at 6 h and 1 year post-transplantation. Donor or recipient identity for each cell was determined by Vireo^26^ based on read pileup results of the most common SNPs from the 1000 Genomes Project (Fig. 3h). All major immune cell subsets could be identified, including T cells, B cells, NK cells, dendritic cells and macrophages (Fig. 3i). In accordance with our flow cytometry data, donor-derived immune cells dominated at 6 h after transplantation, whereas 1 year later recipient-derived cells made up the vast majority of immune cells and donor-derived cells were detectable only within one of the four samples analyzed. The small number of donor-derived cells present within the liver tissue after 1 year of transplantation were of T cell identity (Fig. 3i).

We next examined in more detail the identity and dynamics of NK cells within the transplanted liver. NK cells could be clustered based on their gene expression into two different subsets (Fig. 3j). At 6 h post-transplantation, donor-derived NK cells were the most prevalent cells in cluster 2, whereas recipient-derived NK cells were mainly found in cluster 1. A year after transplantation, recipient cells were present within both defined NK cell clusters. Next, we determined the expression of TR markers expressed within the different NK cell clusters (Fig. 3k). TR markers, including CD69, Eomes, ITGA1 (CD49a) and CXCR6, were mainly expressed by NK cell cluster 2, suggesting that this subset was composed of TR NK cells.

In conclusion, donor-derived TR immune cells within the liver vanish over time and are replaced by recipient-derived immune cells that enter the tissue and acquire TR traits.

### Runx3 contributes to the establishment of human TR NK cells

**Fig. 4:**
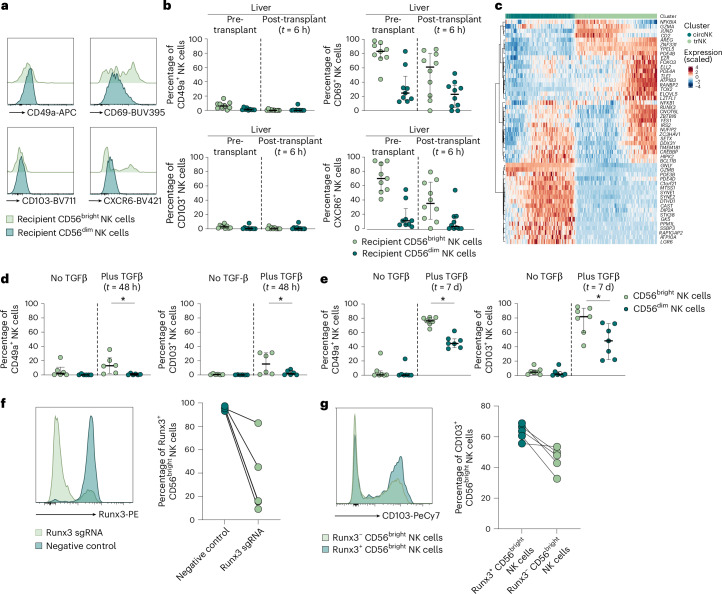
Runx3 partly drives establishment of NK TR phenotype. **a**, Representative histograms of the TR markers, CD49a, CD69, CD103 and CXCR6 in recipient CD56^bright^ (light green) and recipient CD56^dim^ (dark green) liver NK cells at 6 h after liver transplantation. **b**, Percentage of TR marker-positive (CD49a^+^, CD69^+^, CD103^+^ or CXCR6^+^) CD56^bright^ (light green) and CD56^dim^ NK cells (dark green), HLA-recipient^+^ cells in liver tissue pre-transplantation and post-transplantation (*t* = 6 h). Each dot represents one individual donor (*n* = 10). The line indicates the median and the error bars the IQR. **c**, Heatmap of the top 50 RNA-velocity genes (highest velocity fit likelihood) in recipient NK cells (dark green = circulating NK, light green = TR NK) identified by scRNA-seq. Expression is displayed as row-wise *z*-scores. **d**, Percentage of CD49a^+^ and CD103^+^ of sorted CD56^bright^ (light green) and CD56^dim^ (dark green) after 48 h incubation with (right) or without (left) in vitro TGFβ stimulation (*t* = 48 h). Each dot represents one donor (*n* = 6), the line represents the median and the error bars represent the IQR. ^*^P = 0.0312. **e**, Percentage of CD49a^+^ and CD103^+^ of CD56^bright^ (light green) and CD56^dim^ (dark green) after 7 d of incubation with (right) or without (left) in vitro TGFβ stimulation (*t* = 7 d). Each dot represents one donor (*n* = 7), the line represents the median and the error bars represent the IQR. ^*^P = 0.0156. **f**, Representative histogram of Runx3 expression (left) and frequencies of Runx3^+^ CD56^bright^ NK cells (right) after electroporation with Runx3 single guide (sg)RNA (light green) or negative control sgRNA (dark green). Each dot represents one donor (*n* = 5) and the lines connect frequencies of the same donor. **g**, NK cells were electroporated with Runx3 sgRNA and subsequently stimulated with TGFβ for 7 d to determine CD103 expression. Representative histogram of CD103 expression (left) and frequencies of CD103^+^Runx3^−^CD56^bright^ (light green) or Runx3^+^CD56^bright^ (dark green) NK cells (right) are depicted. Each dot represents one donor (*n* = 5) and the lines connect frequencies of the same donor. Statistical significances between frequencies of CD103^+^ and CD49a^+^ NK cells after TGFβ stimulation were assessed using the paired, nonparametric, Wilcoxon’s signed rank test. Two-tailed *P* < 0.05 was considered significant.

To confirm the expression of TR markers observed in single-cell sequencing, we used high-dimensional flow cytometry. As expected, NK cells from PB, except donor-derived CD56^bright^ NK cells, displayed lower expression of TR markers (CD49a, CXCR6, CD69 and CD103) compared to liver-derived NK cells (Supplementary Fig. 3a). Before transplantation, CD56^bright^ NK cells isolated from recipient and donor liver tissue, respectively, displayed high TR molecule expression, whereas 6 h post-transplantation most liver NK cells expressing TR markers were donor-derived and displayed a CD56^bright^ phenotype (Fig. 4a,b). Importantly, a minor population of recipient-derived liver CD56^bright^ NK cells co-expressed CD69 and CXCR6 at 6 h post-transplantation, indicating that recipient-derived cells adapt TR traits early after entering the tissue, whereas no such adaptation was seen within the CD56^dim^ NK cell subset (Fig. 4b and Supplementary Fig. 3b).

In addition, we next compared the expression of intracellular molecules and transcription factors between circulating and TR NK cells. In general, circulating NK cells displayed higher expression of molecules associated with cytotoxicity, including granzyme B (*GZMB*) and granulysin (*GNLY*) (Fig. 4c). In accordance with a recent publication assessing T cell tissue residency, *RUNX3* was among the transcription factors with higher expression in transiently TR NK cells^27,28^ (Supplementary Fig. 3d). To determine the influence of Runx3 on the acquisition of TR molecule expression, we used an in vitro transforming growth factor (TGFβ) stimulation assay. The cytokine TGFβ induces a TR phenotype in multiple human organs including the liver, as well as after in vitro stimulation^29^. On in vitro stimulation, purified human CD56^bright^ NK cells were more responsive to TGFβ than CD56^dim^ NK cells (Fig. 4d,e). To determine whether Runx3 influenced the acquisition of a TR phenotype, we knocked out *RUNX3* expression in primary human NK cells using CRISPR–Cas9 (Fig. 4f). Runx3-deficient CD56^bright^ NK cells exhibited an impaired capability to upregulate CD103 after TGFβ stimulation, indicating a role of Runx3 in the regulation of TR molecule expression in NK cells (Fig. 4g).

In summary, CD56^bright^ NK cells display a higher capability of acquiring TR traits, both in vitro and in vivo, compared to CD56^dim^ NK cells and this is partly regulated by Runx3.

### CD56^bright^ NK cells in lymph fluid display a unique phenotype

**Fig. 5:**
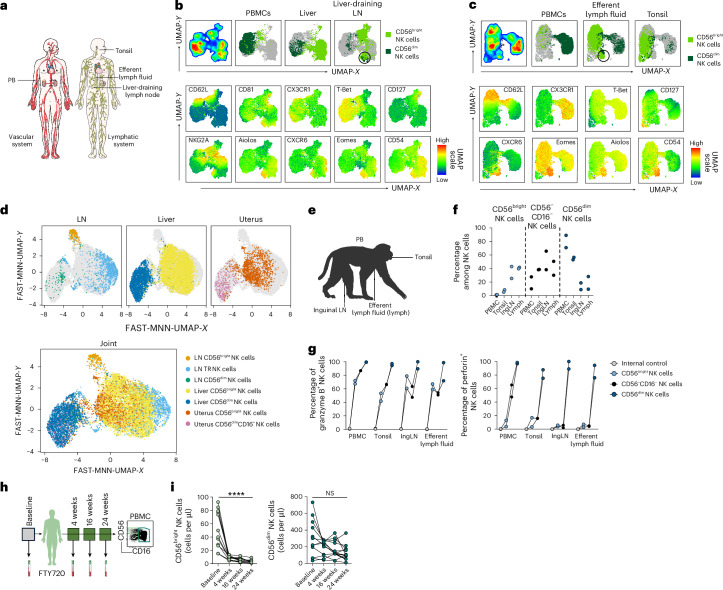
LN-derived NK cells have two distinct phenotypes indicative of different origins. **a**, Sample collection scheme of human lymphoid-derived and vascular-derived samples. **b**, Expression of surface markers analyzed on human PB and liver and liver-draining LN NK cells using flow cytometry. NK cells from PBMCs, liver (both *n* = 5) and liver-derived LNs (*n* = 3) were downsampled, concatenated and visualized by dimensionality reduction (UMAP). Top: UMAPs on total NK cells, separated in cells from PBMCs, liver and liver-draining LNs on CD56^dim^ (dark green) and CD56^bright^ (light green) NK cells. UMAPs in the middle and bottom rows show mean expression of indicated surface molecules. Analyses are based on data from 3–5 individuals with 1,200 events per individual and 15,600 events in total. The circle highlights a population of Aiolos^+^Eomes^+^CXCR6^+^CD54^+^CD62L^−^ NK cells present in liver and liver-draining LNs. **c**, Expression of surface and intracellular markers analyzed on human PB and efferent lymph fluid and tonsil NK cells using flow cytometry. NK cells from four (PBMCs), five (efferent lymph fluid) and three (tonsil) donors were downsampled, concatenated and visualized by dimensionality reduction (UMAP). Top: UMAP maps on total NK cells, separated in cells from PBMCs, efferent lymph fluid and tonsils on CD56^dim^ (dark green) and CD56^bright^ (light green) NK cells. Middle and bottom: UMAPs showing mean expression of indicated surface molecules. Analyses are based on data from 3–5 individuals with 1,000 events per individual and 12,000 events in total. The circle highlights a population of Aiolos^+^Eomes^+^CXCR6^+^CD54^+^CD62L^−^ NK cells present in the efferent lymph fluid. **d**, Publicly available single-cell sequencing dataset from LNs^52^ and uterus^53^ integrated with single-cell sequencing data from liver generated within this study using Seurat function and subsequently plotted using a FAST mutual nearest neighbors (FAST-MNN) UMAP. Top: individual FAST-MNN UMAPs for each tissue highlighting the respective tissue-derived cells, including CD56^bright^ LN-derived NK cells (orange), TR LN-derived NK cells (light blue) and CD56^dim^ LN-derived NK cells (green) (left), CD56^bright^ liver-derived NK cells (yellow), CD56^dim^ liver-derived NK cells (dark blue) (middle), CD56^bright^ uterus-derived NK cells (red) and CD56^dim^ uterus-derived NK cells (pink) (right). Bottom: all NK cell subsets from the distinct tissue together. **e**, Sample collection scheme for macaque efferent lymph fluid. **f**,**g**, Summary data of NK cell subset distribution in indicated macaque tissues (**f**) and granzyme B and perforin expression (**g**) within NK cell subsets. Each dot represents one macaque and the lines connect values from the same animal. Non-NK cell lineage marker-positive cells (CD3^±^CD14^±^CD20^±^CD8a^−^) were used as an internal control. **h**, Collection scheme for PB sample collection from patients with multiple sclerosis before and after start of FTY720 treatment at indicated timepoints. **i**, Absolute numbers of CD56^bright^ (left) and CD56^dim^ (right) NK cells per microliter of blood. Each dot represents an individual donor (*n* = 12). The lines connect values from the same donor among different timepoints. Nonparametric Friedman’s test was used with Dunn’s post-hoc test for multiple-group comparisons for statistical analysis. Only comparisons between baseline and week 24 are depicted within the figure. ^****^P < 0.0001. Two-tailed *P* < 0.05 was considered significant. Illustrations in **a** and **h** from Servier Medical Art (https://smart.servier.com/); panel **e** created using BioRender.com. IngLN, inguinal LN; NS, not significant.

Next, we determined the recirculation patterns of transiently TR CD56^bright^ NK cells by examining the NK cell composition in efferent lymph fluid compared to secondary lymphoid organs and liver (Fig. 5a). Efferent lymph fluid was obtained by thoracic duct cannulation of patients undergoing an interventional radiological examination. Beyond canonical tissue-residency markers, we also included Aiolos and CD54, which have been described as highly expressed in lymphoid-derived NK cells^30^. We noted that two main phenotypes of CD56^bright^ NK cells were present across the studied locations (Fig. 5b,c and Supplementary Fig. 4a–f). First, Aiolos^+^Eomes^+^CXCR6^+^CD54^+^CD62L^−^CD56^bright^ NK cells were present in liver, liver-draining lymph nodes (LNs) and efferent lymph fluid, but not in PB and tonsils. Second, CD62L±Aiolos^low^CXCR6^low^CD54^low^CD56^bright^ NK cells could be found in PB, liver-draining LNs, efferent lymph fluid and tonsils but not in liver. These results suggest that the first phenotype represents transiently TR CD56^bright^ NK cells recirculating from nonlymphoid tissues via afferent lymphatics and LNs and finally to efferent lymphatics before returning to the circulation, because these cells could be found in liver, liver-draining LNs and efferent lymphatics but not in tonsils, which lack afferent lymphatics. The second cluster likely represents PB CD56^bright^ NK cells that directly enter LNs from the circulation and thereafter recirculate. The suggested trafficking routes for the two CD56^bright^ populations outlined above were confirmed using scRNA-seq data (Fig. 5d). Two clusters of CD56^bright^ NK cells were present in LNs, one overlapping with TR NK cell clusters from the liver and uterus (denoted LN TR NK cells) and a second more similar to PB CD56^bright^ NK cells (Fig. 5d and Supplementary Fig. 4g,h). CD56^dim^ NK cells expressed high levels of CX3CR1 protein, a feature of memory T cells with limited lymphatic recirculation^31,32^, and were less prevalent in liver, liver-draining LNs, tonsils and efferent lymphatic fluid (Fig. 5b–d).

We further confirmed our findings that human efferent lymph fluid contains a high proportion of CD56^bright^ NK cells by studying non-human primates. To this end, PB, tonsils, LNs and efferent lymph fluid were collected from two healthy rhesus macaques (RMs; Fig. 5e). In rhesus macaques, NK cells were defined as CD3^−^CD14^−^CD20^−^HLA-DR^−^NKG2A/C^+^CD8α^+^ lymphocytes and further subdivided into CD56^dim^, CD56^bright^ and CD56^−^CD16^−^ NK cells, with the last representing a functional intermediate between CD56^bright^ and CD56^dim^ NK cells^33^. Corroborating findings from humans, CD56^dim^ NK cells were dominant in macaque PB, less frequent in tonsils and rare in LNs and efferent lymph fluid. In contrast, CD56^bright^ NK cells, were highly abundant in LNs and efferent lymph fluid compared to PB (Fig. 5f). Macaque CD56^bright^ NK cells, regardless of localization, expressed intermediate amounts of granzyme B, yet the amounts of granzyme B were higher within CD56^dim^ NK cells and the expression of perforin was primarily confined to CD56^dim^ NK cells (Fig. 5g).

In summary, these results identify lymphatics as the primary route of tissue egress for transiently TR CD56^bright^ NK cells from tissues. It further shows that the cells retain a consistent tissue-imprint phenotype throughout their recirculation.

### LN egress blockade decreases number of CD56^bright^ NK cells in blood

To further assess recirculation patterns of NK cells, we studied the NK cell subset composition in PB of patients with multiple sclerosis before and after FTY720 treatment (Fig. 5h). FTY720 is a sphingosine-1-phosphate receptor modulator which sequesters lymphocytes in LNs^34^. As expected, both the absolute number and the frequency of CD56^bright^ NK cells in PB decreased significantly after FTY720 treatment. In contrast, CD56^dim^ NK cell numbers remained stable (Fig. 5i). Furthermore, the proportion of CD56^dim^ NK cells among total NK cells increased after treatment, whereas the frequency of CD56^bright^ NK cells significantly decreased over time (Supplementary Fig. 4i). Moreover, the expression of CCR6 and CCR7, two chemokine receptors related to homing to mucosal tissues and secondary lymphoid tissues, decreased during FTY720 treatment within CD56^dim^ and CD56^bright^ NK cells, whereas the fraction of CX3CR1-expressing NK cells increased (Supplementary Fig. 4j).

In summary, the high abundance of CD56^bright^ NK cells in the lymphatic fluid and the decrease of CD56^bright^ NK cells in PB after blocking LN egress further support that CD56^bright^ NK cells continuously recirculate from the tissue via the lymphatic system at steady state.

### Cytotoxic NK cells enter LNs on inflammatory conditions

**Fig. 6:**
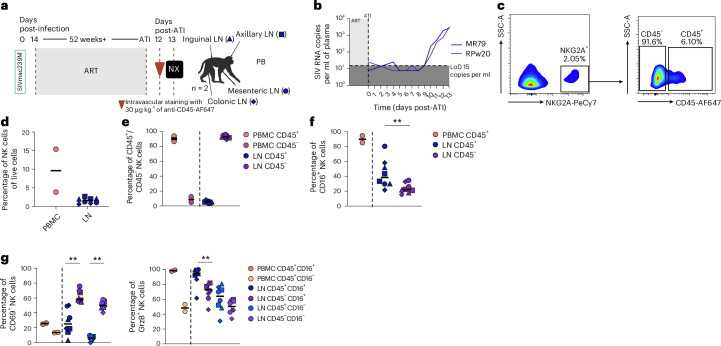
Cytotoxic NK cells enter the LNs on infection. **a**, Schematic overview of the experimental SIV infection. Two RMs were infected with SIVmac239M and started on day 14 post-infection on daily subcutaneous ART for >52 weeks. Subsequently ART was interrupted (analytical treatment interruption (ATI)) for a total of 12 d and animals were intravascularly injected with anti-CD45-AF647 antibody before necropsy on day 13. PB and LNs from four distinct sites (axial, mesenterial, colonic and inguinal) were harvested and analyzed. **b**, SIV RNA copies per ml of plasma of RMs after being ATI tested. Each line depicts the number of SIV RNA copies in one of the respective animals. The limit of detection (LoD) was 15 copies per ml. **c**, Gating strategy to identify NK cells defined as NKG2A^+^ cells (left) and subsequent identification of cells positive and negative for intravascular CD45 labeling in LN tissue (right). **d**, Frequency of NK cells in PBMCs and LNs. Each dot represents one animal (PBMCs) (*n* = 2) or one LN site from one animal (*n* = 8). The symbols indicate the different LN sites and the line represents the median. **e**, Frequency of CD45^+^ and CD45^−^ PBMC-derived NK cells (orange and pink) and CD45^+^ and CD45^−^ LN-derived NK cells (blue and purple). Each dot represents one animal (PBMC) (*n* = 2) or one LN site from one animal (*n* = 8). The symbols indicate the different LN sites and the line represents the median. **f**, Frequency of CD16^+^ NK cells determined in CD45^+^ PBMCs (orange), CD45^+^ LN-derived (blue) and CD45^−^ LN-derived (purple) cells. Each dot represents one animal (PBMCs) (*n* = 2) or one LN site from one animal (*n* = 8). The symbols indicate the different LN sites and the line represents the median. ^**^P = 0.0078. **g**, Frequencies of CD69^+^ and granzyme B^+^ NK cells depicted for PBMC-derived CD45^+^CD16^+^ (orange) and CD45^+^CD16^−^ (yellow) cells as well as for LN-derived CD45^+^CD16^+^ (dark blue), CD45^−^CD16^+^ (dark purple), CD45^+^CD16^−^ (light blue) and CD45^−^CD16^−^ (light purple) cells. Each dot represents one animal (PBMCs) (*n* = 2) or one LN site from one animal (*n* = 8). The symbols indicate the different LN sites and the line represents the median. ^**^P = 0.078. Statistical differences were assessed using the paired, nonparametric, Wilcoxon’s rank sum test for comparing LN CD45^+^-matched and LN CD45^−^-matched groups. Two-tailed *P* < 0.05 was considered significant. Panel **a** created using BioRender.com. GrzB, granzyme.

Results this far indicate that human CD56^dim^ NK cells are constrained to vasculature and possess a lower potential to adapt to a tissue environment at steady state. To determine whether CD56^dim^ NK cells would be able to enter LNs during inflammation, we used chronically simian immunodeficiency virus (SIV)-infected macaques that had been on antiviral treatment for 7 months and up to 12 months, successfully suppressing SIV (Fig. 6a and Supplementary Fig. 5a). On treatment interruption the virus rebounds (Fig. 6b). First, we longitudinally mapped NK cell subsets in peripheral blood mononuclear cells (PBMCs) and LNs over time, before antiretroviral therapy (ART) interruption and during ART interruption as well as after re-treatment (Supplementary Fig. 5). The fraction of CD56^dim^ NK cells was increased in LNs 60 d (day 280) after ART interruption (Supplementary Fig. 5e), but returned to low levels after re-treatment (day 310). This shows that CD56^dim^ NK cell entry into LNs is dependent on the presence of the virus.

To dynamically study this, we focused on the earlier 2-week timepoint after ART interruption. Animals were injected with an i.v. CD45 antibody 12 d after treatment interruption and sacrificed 24 h after labeling. NK cells were defined as CD3^−^NKG2A/C^+^ cells and subsequently gated into CD45^+^ and CD45^−^ cells according to the staining with the injected i.v. CD45 antibody (Fig. 6c). The combined median frequencies of NK cells within analyzed LN (colonic, inguinal, axillary and mesenterial) was 1.65% (Fig. 6d). As expected, NK cells in PB displayed a high frequency of i.v. CD45^+^ labeling but a small subset of i.v. CD45^+^ NK cells could be identified in LNs suggesting homing that had occurred in the preceding 24 h (Fig. 6e). CD16 expression was almost twice as high on i.v. CD45^+^ LN-derived NK cells compared to CD45^−^ LN-derived NK cells (Fig. 6f). This small population of recently entered CD16^+^ i.v. CD45^+^ NK cells also displayed the highest expression of granzyme B within LNs, yet these cells displayed lower CD69 compared to CD45^−^ LN NK cells (Fig. 6g). Thus, these results indicate that cytotoxic NK cells can enter the LN tissue parenchyma during inflammatory conditions, whereas cytotoxic NK cells are constrained to vasculature at a steady state.

Together, these findings identify CD56^bright^ NK cells as the transiently TR and recirculating NK cell subset at steady state, while revealing that CD56^dim^ cytotoxic NK cells gain tissue access during inflammation, suggesting distinct and context-dependent programs of NK cell tissue adaptation.

## Discussion

Our knowledge of the importance of NK cells in tissues has increased significantly in recent years, but details on how human NK cell subsets access tissue, form residency or recirculate have remained scarce. Here we dissected the distinct characteristics of NK cells across tissues, their access to tissue parenchyma at steady state and inflammatory conditions, as well as the dynamic behavior and recirculation patterns of NK cell populations. Our findings provide a new framework for NK cell subset trafficking behavior in humans.

CD56^bright^ NK cells expressing high levels of CD49a, CXCR6, CD69 and/or CD103 have been shown to be the predominant NK cell subset within human tissues^1,3,7–14,18,19^ and we confirm these results across multiple human tissues. In addition to the expression of shared tissue-residency markers such as CD69, CD56^bright^ NK cells displayed TR traits that reflected the tissue microenvironment from which they originated, for example, expression of cutaneous lymphocyte-associated antigen within skin and sAT-resident NK cells.

In addition to surface markers, several transcriptional factors have been identified that dictate tissue-residency programs^35,36^. TR T cells have been previously described to express FOXO3 and AREG, which subsequently mediate tissue repair and adaptive responses^37,38^. Within our liver transplantation cohort, transiently TR liver NK cells expressed higher levels of FOXO3 and AREG compared to circulating NK cells. Whether these molecules mediate the same effects in TR NK cells as they do in T cells is currently unknown. However, CXCR6^+^ NK cells from the liver have been previously described to mount adaptive responses on antigen challenge^39^, indicating that those molecules might have similar functions. We identified *RUNX3* as an additional transcription factor upregulated by transiently liver-resident NK cells. *RUNX3* has been previously shown to mediate the establishment of a TR T cell phenotype^27,28^ and, using CRISPR–Cas9 knockout experiments, we now describe a new role of *RUNX3* in the establishment of the transiently TR phenotype observed in CD56^bright^ NK cells. As lack of *RUNX3* was insufficient to completely abrogate the upregulation of TR molecules on CD56^bright^ NK cells, additional transcription factors likely contribute to the establishment of a TR NK cell phenotype.

It has been reported that TR T cells can reside in the human liver for years after liver transplantion^40^, arguing for the long-term maintenance of these cells within tissues. TR T cells have also been described to be long lived after intestine and lung transplantation in humans^41,42^. However, NK cell and mucosal-associated invariant T cell (MAIT) populations from other organs, for example, the uterus, appear to have a more transient TR profile and to be replaced by infiltrating immune cells from the PB over time^43,44^. Our results suggest a similar pattern for NK cells from the liver with a transient TR phenotype and a fast turnover rate. Not only NK cells but also regulatory T (T_reg_) cells have been previously reported to display a transient TR phenotype with activated T_reg_ cells entering the tissue, upregulating TR molecules but subsequently recirculating^16^. Within our liver transplantation cohort, we observed an almost complete replacement of immune cell populations, including NK cells, over time. Recipient cells infiltrated the liver and acquired TR marker expression as early as 6 h after transplantation. Yet, a few cells in a minority of samples were still donor derived 1 year after transplantation. Those HLA-donor^+^ cells were mostly T cells as identified by flow cytometry and scRNA-seq. It is currently unclear what regulates the tissue-residency longevity and whether there are distinct TR mechanisms for transiently compared to long-term TR cells. Factors to consider include loss of NK cells in tissues due to apoptosis or a natural turnover at a specific rate due to different lifespans of NK cells and T cells. These are challenging to measure in vivo and represent a limitation of the current study, adding uncertainty in interpreting tissue-residency duration and replacement dynamics. Another possible explanation for the different kinetics could be the intraparenchymal localization of TR immune cells within different organs, with the lung and gut having a higher fraction of intraepithelial CD69^+^CD103^+^ lymphocytes whereas the liver and uterus mostly have parenchymal CD69^+^ lymphocytes. In addition, TR NK cells across different organs exhibited distinct phenotypes that were mediated by the different tissue microenvironments. Those might influence the spatial localization and overall turnover rate of these cells within different human tissues. Importantly, all patients undergoing liver transplantation received standard immunosuppression consisting of tacrolimus, basiliximab, methylprednisolone, prednisolone and mycophenolate mofetil. We cannot exclude that such immunosuppressive treatment has an impact on the replenishment of TR immune cells after liver transplantation. However, we observed a complete replenishment of recipient NK cells expressing TR molecules 1 year after transplantation, which argues against any long-term effect on the migration behavior of NK cells or their capability of adopting TR traits.

As the MISTRG humanized mouse model efficiently supports human NK cell development^22^, we investigated NK cell subset localization within tissues using this model. The human NK cell subset composition in MISTRG mice was similar to human tissues, with CD56^+^CD16^+^ NK cells predominant in blood and CD56^+^CD16^−^ NK cells more prevalent in tissues. In contrast to CD56^+^CD16^+^ NK cells, CD56^+^CD16^−^ NK cells had access to the tissue parenchyma at steady state, explaining the high abundance of these cells within tissues. Previous studies in RMs also highlighted kinetic differences between CD56^+^ and CD16^+^ NK cells within the vasculature, with CD16^+^ NK cells exhibiting constant vascular circulation whereas CD56^+^ NK cells resided for shorter time periods in the vascular system^45^. CD56^dim^ NK cells dominated within afferent and efferent venous blood of the human liver, confirming our humanized mice results that those cells do not extravasate at steady state.

CD56^bright^ NK cells were less abundant in the efferent and afferent blood of the liver compared to CD56^dim^ NK cells and CD56^bright^ NK cell frequencies did not vary between afferent and efferent. CD56^bright^ NK cells were more present in the lymph fluid and inhibition of LN egress by FTY720 led to a drop of CD56^bright^ NK cells within the circulation. Overall, these results imply that, similar to the recirculation of T cells, the lymphoid system represents the main route of recirculation for CD56^bright^ NK cells.

Interestingly, NK cells within the liver, liver-draining LNs and efferent lymph fluid displayed a consistent phenotype with Aiolos, Eomes, CXCR6 and CD54 expression, indicative of a conserved phenotype throughout the recirculation route. CD54^+^ NK cells have been recently identified in tonsils and lymphoid tissue, with CD54^high^ expressing cells displaying a TR phenotype and a higher capacity to produce interferon-γ^9,46^. Our identification of liver-derived CD56^bright^ NK cells expressing CD54 in combination with TR markers suggests that CD54 expression might be a common feature of TR NK cells across multiple human tissues. However, whether CD54 has a functional role in the formation or retention of TR cells needs to be studied further. In addition, we observed a second cluster of NK cells within LNs with high expression of the LN-homing molecule CD62L. Collectively these results indicate the presence of two distinct NK cell phenotypes within human LNs, representing recirculating NK cells from peripheral tissues as well as NK cells entering and homing to lymphoid tissue exclusively.

A third of all liver-derived NK cells displayed a CD56^dim^ phenotype. In this context, it was shown that liver CD56^dim^ NK cells resemble blood CD56^dim^ NK cells, with low expression of TR markers^47^. Why CD56^dim^ NK cells are confined to the vasculature at steady state is not completely understood. Studies of CD8 T cells have demonstrated that CX3CR1^+^ cytotoxic, memory CD8 T cells^48^ have limited access to the extravascular space and are not recirculating via the lymphatics^31,32^. Corroborating these studies, we showed that CX3CR1 was mainly expressed by CD56^dim^ NK cells. In addition, CD56^dim^ NK cells expressed higher levels of cytotoxic effector molecules, like granzyme B and perforin, compared to CD56^bright^ NK cells. CD56^bright^ NK cells exhibited a lower expression of cytotoxic effector molecules and were implicated as playing an important role in tissue homeostasis^4^, potentially also explaining their access to the tissue parenchyma during steady-state conditions. However, on an initial inflammatory event, CD56^dim^ NK cells can be recruited to inflammatory sites^49–51^ and our RM data indicate the presence of CD16^+^ NK cells within the tissue parenchyma of LNs on SIV infection.

Overall, our study provides new insights into NK cells in tissues, their recirculation kinetics and patterns, building a mechanistic framework for NK cell tissue residency.

## Methods

### Study cohorts and ethics

PB and disease-unaffected duodenum tissue samples were obtained from patients with intraductal papillary mucinous neoplasms undergoing pancreatoduodenectomy. PB samples were collected from healthy donors. Liver tissue samples were obtained from organ donor livers not used for transplantation, uterine tissues were taken during hysterectomies performed for benign reasons and tonsils were obtained from patients with obstructive sleep apnea syndrome. Human CD34^+^ cells for the generation of humanized mice were obtained from umbilical cord blood. Liver biopsies and PB samples from patients undergoing liver transplantation were collected at different timepoints before and after transplantation. Liver-draining LNs were collected from patients undergoing distal pancreatectomy. All of the above samples were collected at the Karolinska University Hospital, Stockholm, Sweden. Afferent and efferent venous blood samples from human liver, liver tissue and PB samples were taken during open abdominal surgery or wedged hepatic venous pressure measurement at the Hannover Medical School, Hannover, Germany. Efferent lymph fluid and PB were taken from patients with leakages of their thoracic duct, lymphatic disorders or trauma to the duct at the University of Pennsylvania and the Children’s Hospital of Philadelphia, as previously described^54^. PB samples were taken from patients with relapsing–remitting multiple sclerosis^55^ before (baseline) and 4 weeks, 16 weeks and 24 weeks after FTY720 (fingolimod) treatment at the Department of Neurology, University of Muenster, Germany (NCT02325440). Written informed consent was obtained from all patients described above. The studies were approved by the regional ethics committee, Stockholm County, Stockholm, Sweden, Hannover Medical School, the German competent authority (Federal Institute for Drugs and Medical Devices) and the institutional review board of the University of Pennsylvania, Children’s Hospital of Philadelphia, and performed in accordance with the Declaration of Helsinki.

### Preparation of human tissue, blood and efferent lymph fluid

Immune cells were isolated from PB and efferent lymph fluid using Ficoll Hypaque gradient centrifugation (Lymphoprep) and washed in phosphate-buffered saline (PBS). Duodenum, uterine and liver tissues, as well as liver-draining LNs, were cut into small pieces, transferred into Roswell Park Memorial Institute (RPMI) 1640 medium (Thermo Fisher Scientific) and digested for 30 min at 37 °C with collagenase II (0.25 mg ml^−1^, Sigma-Aldrich) and DNase (0.2 mg ml^−1^; Roche). Tonsils were cut and mashed through a 100-μm nylon cell strainer into complete RPMI medium and 1 mM l-glutamine (Thermo Fisher Scientific). All tissue-derived cell suspensions were additionally filtered through a 70-μm cell strainer. Sample sizes used for downstream experiments were determined based on sample availability with no previous power analysis. No blinding or randomization was performed for downstream experiments.

### Collection and preparation of RM samples

RMs (*Macaca mulatta*) of Indian origin were obtained from the University of Pennsylvania. The number of animals used for individual experiments was based on comparable numbers reported in previous publications^45,56^. No blinding or randomization was used for any downstream analysis. Animals were anesthetized and cannulated for PB and efferent lymph fluid sampling. LNs and tonsils were obtained during necropsy. PB, efferent lymph fluid and tissue samples were processed in the same way as the human samples described above. In addition, this study included specific pathogen-free RMs single housed in an animal BSL-2 facility at Emory National Primate Research Center, Atlanta, GA. RMs were infected intravenously with 10,000 IU of barcoded SIVmac239M and started, at day 14 post-infection, on a daily subcutaneous ART regimen of emtricitabine (FTC, 40 mg kg^−1^), tenofovir disoproxil fumarate (5.1 mg kg^−1^) and dolutegravir (2.5 mg kg^−1^), which was maintained until the indicated timepoints of treatment interruption. For seven of the animals, daily ART treatment was reintroduced at 60 d post-treatment interruption. PB and LNs were collected at different timepoints throughout infection and processed directly as described below. Animals used for αCD45 labeling received one intravenous infusion of the αCD45-AF647 (clone ITS_rhCD45; purchased from Nonhuman Primate Reagent Resource) at day 12 post-treatment interruption, diluted in 5 ml of sterile Hanks’ buffered salt solution at a dose of 30 μg kg^−1^. Sample collection was performed 1 d post-infusion, at the time of necropsy. All animals were monitored for 13 d before necropsy. Plasma, PBMCs and LNs from different areas (axillary, inguinal, mesenteric and colonic) were collected 1 d post-intravascular staining and processed as previously described^56–59^. Briefly, blood was centrifuged to separate the plasma, which was aliquoted and frozen at −80 °C until downstream analysis. PBMCs were isolated from whole blood by density gradient centrifugation. SIV plasma viral loads were measured as previously described with a limit of detection of 15 copies per ml^60^. LNs were homogenized and passed through a 70-μm cell strainer to isolate lymphocytes. Mononuclear cells were counted for viability using a Countess II Automated Cell Counter (Thermo Fisher Scientific) with Trypan Blue stain. All samples were processed, stained and analyzed by flow cytometry immediately after collection. The remaining mononuclear cells were cryopreserved and stored in liquid nitrogen for downstream assays. Multiparametric flow cytometry was performed using standard procedures on fresh PBMCs and mononuclear cells derived from LNs using anti-human monoclonal antibodies previously shown to be crossreactive in RMs^59,61,62^. Gated NK cell subpopulations with <15 events in the final population were excluded in the analysis. All macaque studies were approved by the Institutional Animal Care and Use Committee at the University of Pennsylvania and all procedures were conducted in accordance with the Animal Welfare Act and other US federal statutes and regulations relating to animals. Animal care facilities at Emory National Primate Research Center are accredited by the US Department of Agriculture and the Association for Assessment and Accreditation of Laboratory Animal Care International.

### Intravascular cell labeling and processing of MISTRG tissue sample

MISTRG mice homozygous for the human genes encoding macrophage colony-stimulating factor, interleukin (IL)-3, granulocyte–macrophage colony-stimulating factor, signal regulatory protein α and thyroid peroxidase, on the Rag2^−/−^Il2rg^−/−^ background, have been previously described^22^. The number of animals used for individual experiments was based on comparable numbers reported in previous publications^25,63^. MISTRG mice were housed in individually ventilated cages under specific pathogen-free conditions without any prophylactic antibiotics. Both male and female mice were used for the experiments. Mice were housed on a dark-to-light cycle of 12 h, 22 °C ambient temperature and 50 ± 10% humidity. No blinding or randomization was used for any downstream analysis. To generate mice with a human immune system, newborn MISTRG mice were transplanted with 5 × 10^4^ to 1 × 10^5^ human CD34^+^ cells (isolated from umbilical cord blood) by intrahepatic injection without any preconditioning by irradiation, as previously described^25^. For the labeling of vascular CD45^+^ immune cells, 3-month-old MISTRG mice engrafted with CD34^+^ cells received an intravenous injection of 2 μg of PE-labeled anti-human CD45 antibody (BioLegend, cat. no. HI30) as described^25,63–65^. Then, 5 min after antibody injection, mice were sacrificed and the organs harvested for flow cytometry. PB was collected into heparin-containing tubes (Sigma-Aldrich). Liver, kidney, uterus and AT were harvested into complete RPMI medium. All tissues were manually mashed and filtered through a 70-μm cell strainer. Erythrocytes were eliminated using erythrocyte lysis buffer according to the manufacturer’s instructions (RBC Lysis Buffer, Thermo Fisher Scientific). All mouse experiments were approved by the Linköping Animal Experimentation Ethics Committee and performed in accordance with local guidelines.

### CRISPR–Cas9 knockout

PB NK cells were isolated from healthy donors using Ficoll Hypaque gradient centrifugation and the human NK cell isolation kit (Miltenyi Biotec) according to the manufacturer’s instruction. Isolated NK cells were cultured overnight in RPMI medium with 10% fetal bovine serum (FBS) and 10 ng ml^−1^ of IL-15 (Peprotech) before undergoing CRISPR–Cas9 knockout. Runx3-crRNA (DesignID#Runx3.1AD, sequence: ACAGTCACCACCGTACCATCCGG, IDT), and negative control CRISPR RNA (crRNA) (negative control crRNA#1, IDT, cat. no. 1072544) were complexed at an equal molar ratio (100 µM) with transactivating crRNA (IDT) for 5 min at 95 °C. Cas9 nuclease (IDT) was diluted to a final concentration of 6 µM and incubated with RNA complexes and ALT-R CRISPR enhancer (IDT) for 15 min at room temperature. Electroporation was carried out using the P3 Primary Cell 96-well Nucleofector Kit from Lonza Technologies. Isolated NK cells were washed twice with PBS to remove residual medium, resuspended in supplemented P3 Primary Cell Nucleofector Solution. NK cells, 1 × 10^6^, were subsequently plated per well of the 96-well Nucleocuvette Plate and the respective RNA complexes added. Wells were transferred to the Lonza 4D Nucleofactor and pulsed using program CM137. Cells were subsequently rested, recovered with fresh RPMI medium with 10% FBS and transferred to a 96-well plate and incubated overnight. TGFβ (5 ng ml^−1^; Peprotech) was added after overnight incubation and cells were incubated for 7 d and subsequently stained for multicolor flow cytometry.

### Staining for multicolor flow cytometry

HLA molecules used for donor or recipient HLA molecule-specific staining are defined in Supplementary Table 1. All extracellular staining was performed for 30 min at room temperature. Dead cells were excluded using fixable LIVE/DEAD Aqua, Amcyan or Green dead cell stain kits (Life Technologies) or Fixable Viability Stain 700 (BD Biosciences). For intracellular staining, cells were fixed and permeabilized using Fixation/Permeabilization buffer (eBioscience) and subsequently stained for 30 min at room temperature in Perm/Wash buffer (eBioscience) using intracellular antibodies (Supplementary Table 2). Alternatively, samples underwent fixation and permeabilization with Tonbo Foxp3/Transcription Factor Staining Buffer Kit (Cytek Bio) for 45 min at 4 °C. Samples were acquired on a BD LSR Fortessa equipped with five lasers (BD Biosciences), BD LSRII flow cytometer (BD Biosciences), BD FACSymphony A5 equipped with five lasers (BD Biosciences) or Navios flow cytometer (Beckman Coulter). A detailed list of all antibodies used within the study is provided in Supplementary Table 2.

### NK cell sorting and TGFβ stimulation

PB NK cells were isolated from healthy donors using Ficoll Hypaque gradient centrifugation and the human NK cell isolation kit according to the manufacturer’s instructions. NK cells were stained using CD56-Pe-Cy7 (BD Biosciences) and CD16-BV421 (BioLegend) and CD56^bright^CD16^−^ and CD56^dim^CD16^+^ NK cells were sorted using a BD FACS Aria Fusion (BD Biosciences). Cells were incubated overnight at 37 °C, with 5% CO_2_ in RPMI with 10% FBS and 10 ng ml^−1^ of IL-15. After overnight incubation, TGFβ (5 ng ml^−1^) was added to the respective wells and NK cells were incubated for 48 h and analyzed for the expression of TR markers. Alternatively, bulk isolated NK cells were stimulated with TGFβ (5 ng ml^−1^) for 7 d and analyzed for the expression of TR markers.

### Flow cytometry data analysis

Standard flow cytometry data analysis was performed using FlowJo v.10.4.2. or Kaluza software 1.5a (Beckman Coulter). Cells from human samples were defined as live, CD45^+^ leukocyte singlets. Out of these, NK cells were gated as CD14^−^CD15^−^CD19^−^CD3^−^CD56^+^ cells, excluding CD127^+^CD94^−^ ILCs. CD56^dim^ NK cells were defined as CD56^dim^CD16^+^ and CD56^bright^ NK cells as CD56^bright^CD16^−/+^ cells. RM-derived samples were analyzed as previously described^66^. In brief, from live CD45^+^ single cells, NK cells were defined as CD3^−^CD14^−^CD20^−^CD8a^+^NKG2A/C^+^ cells excluding HLA-DR^high^ cells. According to the expression of CD56 and CD16, three NK cell subsets, CD56^dim^ (CD56^−^CD16^+^), CD56^bright^ (CD56^+^CD16^−^) and CD56^−^CD16^−^, were defined. Overall, NK cells from MISTRG mice were defined in the same way as NK cells from human samples. In contrast to human NK cells, all NK cells express high levels of CD56 in the MISTRG mice, resulting in two main NK cell subsets: CD56^+^CD16^−^ and CD56^+^CD16^+^ cells. NK cells residing in vasculature were differentiated from TR NK cells through positive staining for the anti-CD45-PE antibody that had been used for i.v. labeling of vascular leukocytes. UMAP analyses were performed using the UMAP FlowJo plugin (v.3.1) and ran on downsampled fcs files of NK cells per individual for comparability. NK cell cluster generation was performed using the FlowJo plugin Phenograph (v.3.0). Analysis and visualization of surface marker expression on NK cell subsets were further performed in R Studio using the packages ggplot2 (v.3.3.2), reshape2 (v.1.4.4), tidyr (v.1.1.1), viridis (v.0.5.1), dplyr (v.1.0.1) and pheatmap (v.1.0v12).

### ScRNA-seq of liver biopsy samples

Frozen liver cell suspensions were thawed, filtered through a 40-µm mesh and sorted for live (LIVE/DEAD Aqua, Life Technologies) CD45^+^ cells (BioLegend, cat. no. CD45 BV711) using a SONY MA900 cell sorter. A maximum of 12,500 cells per sample were loaded for scRNA-seq. RNA-seq libraries from enriched cells were prepared using the 10x Genomics Gene Expression v.3.1 library kit according to the manufacturer’s protocol. For sequencing on the DNBSEQ platform, libraries were converted to circular single-stranded DNA using the MGIEasy Universal Library Conversion Kit (MGI Tech).

### ScRNA-seq data analysis

FASTQ files were demultiplexed using deML v.1.1.3^67^. After demultiplexing, reads from each of the eight samples were quality filtered, aligned to the hg38 reference genome and quantified based on GENCODE v.35 gene annotations, using the zUMIs pipeline v.2.9.4f^68^. CellBender 0.1.0^69^ was employed to remove empty droplets and background noise and Solo 1.0^70^ to eliminate doublets.

Individual cell identities were assigned by first summarizing read counts on reference or alternative alleles using cellsnp-lite 1.2.0^71^, based on ~7.4 million common variants with an allele frequency >5% in the 1000 Genomes project phase 3 release. Subsequently, based on the genotyping results, cells were assigned to eight genetic identities (--nDonor 8) using Vireo 0.4.2^26^. A final 27,063 cells with >200 detected genes and <20% mitochondrial content and successful donor or recipient assignment were retained for further analysis. Inferred genetic identities were visualized using the ggalluvial 0.12.3 package^72^.

Expression matrices from the eight samples were merged, log(normalized) and the normalized expression data of the top 2,000 most variable features (selection.method = ‘vst’) were scaled in Seurat 4.1.0^73^. Principal component analysis dimensionality reduction was subsequently performed on the scaled data (npcs = 20). Expression data from different samples were integrated using Harmony 0.1^74^. UMAP and clustering were then performed on the integrated harmony reduction. Azimuth human liver and PBMC references (https://azimuth.hubmapconsortium.org/references) were used to annotate major cell types in Seurat. Within NK cells, we performed reclustering, repeating the same procedure and manual annotation of NK subtypes.

To conduct RNA-velocity analysis, we utilized Velocyto command line tool 0.17.15^75^ to count spliced and unspliced reads using the BAM file generated from zUMI. To prevent counting reads on expressed repetitive sequences, we supplied Velocyto with RepeatMasker repeats in GTF format, obtained from the University of California Santa Cruz table browser (last accessed June 2022). The count matrices were then imported into scVelo^76^ for filtering and normalization, retaining the top 2,000 highly variable genes with a minimum of 30 spliced or unspliced counts (n_top_genes = 2000 and min_shared_counts = 30) for further analysis. The full splicing kinetics were recovered and RNA velocity was calculated using a dynamic model. Velocity was then regularized by combining a velocity kernel (weight = 0.8) with a connectivity kernel (weight = 0.2) using the CellRank package^77^. The top 50 putative driver genes (excluding ribosomal and sex-linked genes) with the highest fit likelihood were identified and latent time was computed using the latent_time() function. The top 50 putative driver genes were plotted using the Complex Heatmap package (v.2.18.0)^78^. Annotated NK cells from two publicly available datasets from single-cell sequencing of human uterus tissue^53^ and human LNs^52^, as well as our generated liver biopsy single-cell sequencing dataset, were merged using the merge () function in the Seurat package (v.4.1.0), resulting in a combined dataset of 16,743 cells. Batch correction and integration across donors were conducted using the bachelor package’s FastMNNIntegration() function via IntegrateLayers() in Seurat and dimensionality reduction was performed with UMAP on the integrated space (RunUMAP(), method = ‘uwot’). Clustering was carried out using mutual nearest neighbor (MNN) graph construction (FindNeighbors()) and modularity-based clustering (FindClusters(), resolution = 0.2, method = 4). MNN is particularly effective at preserving local neighborhood structure and reducing batch effects for integrating heterogeneous single-cell data^79^. Cell-type distributions and gene expression patterns of surface markers and transcription factors were visualized using dittoDimPlot() from the dittoSeq package (v.1.18.0)^80^ and DotPlot_scCustom() with the scCustomize (v.3.0.1)^81^ and viridis packages (v.0.6.5).

### Statistical analysis

Statistical analysis was conducted using GraphPad Prism v.7 (GraphPad software). Two-tailed and nonparametric Wilcoxon’s matched pairs, signed rank test was used to compare two matched groups. The nonparametric Kruskal–Wallis test for unmatched and Friedman’s test for matched samples were used in combination with Dunn’s post-hoc test for multiple-group comparisons. *P* < 0.05 was considered significant: ^*^P  < 0.05; ^**^P  < 0.01; ^***^P  < 0.001. Where indicated, the *z*-score of median fluorescence intensity or patient clinical parameters were calculated as follows: *z* = ((*x* − *μ*))/*σ*, where *x* = raw score, *µ* = mean of sample distribution and *σ* = s.d.

### Reporting summary

Further information on research design is available in the Nature Portfolio Reporting Summary linked to this article.

## Online content

Any methods, additional references, Nature Portfolio reporting summaries, source data, extended data, supplementary information, acknowledgements, peer review information; details of author contributions and competing interests; and statements of data and code availability are available at 10.1038/s41590-025-02290-9.

## Supplementary information

Supplementary Information Supplementary Figs. 1–5 and Tables 1 and 2.

Reporting Summary

## Data Availability

Sequencing data have been deposited in the Gene Expression Omnibus database under accession no. GSE246994.

## Code availability

Code used to analyze scRNA-seq data within this manuscript is available via figshare at 10.6084/m9.figshare.29908631.v1 (ref. ^82^).
