# Supplementary material for: Transient tissue residency and lymphatic egress define human CD56bright NK cell homeostasis
Source: Nat Immunol. 2025 Oct 14;26(11):2004–15. doi: 10.1038/s41590-025-02290-9 (PMC12571907; doi:10.1038/s41590-025-02290-9)
Supplement: Supplementary file 1 — Supplementary Figs. 1–5 and Tables 1 and 2. [file 41590_2025_2290_MOESM1_ESM.pdf]

nature immunology

Supplementary information

https://doi.org/10.1038/s41590-025-02290-9

Transient tissue residency and lymphatic egress define human CD56^{bright} NK cell homeostasis

In the format provided by the authors and unedited

Supplementary Figure 1

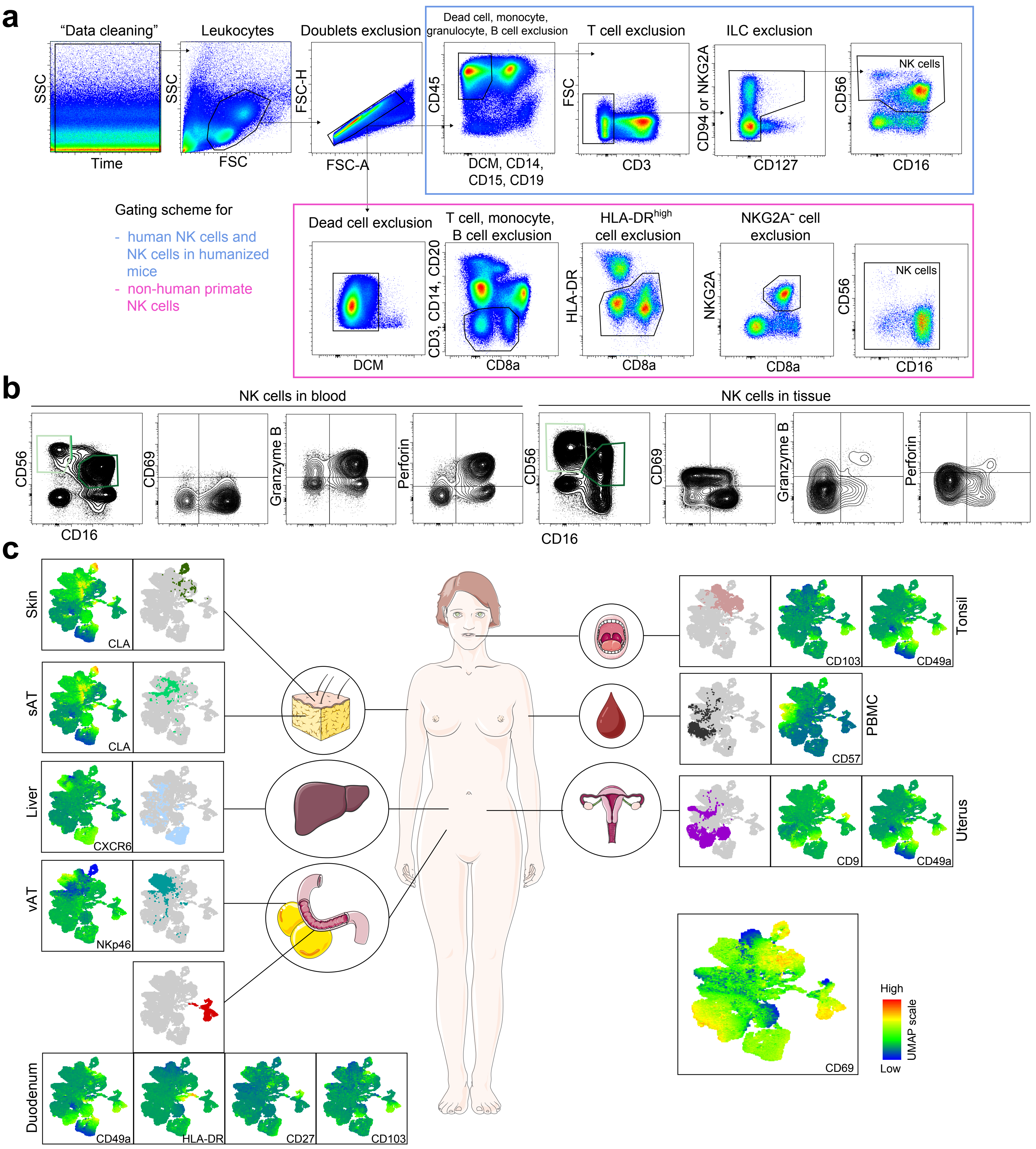

Supplementary Figure 2

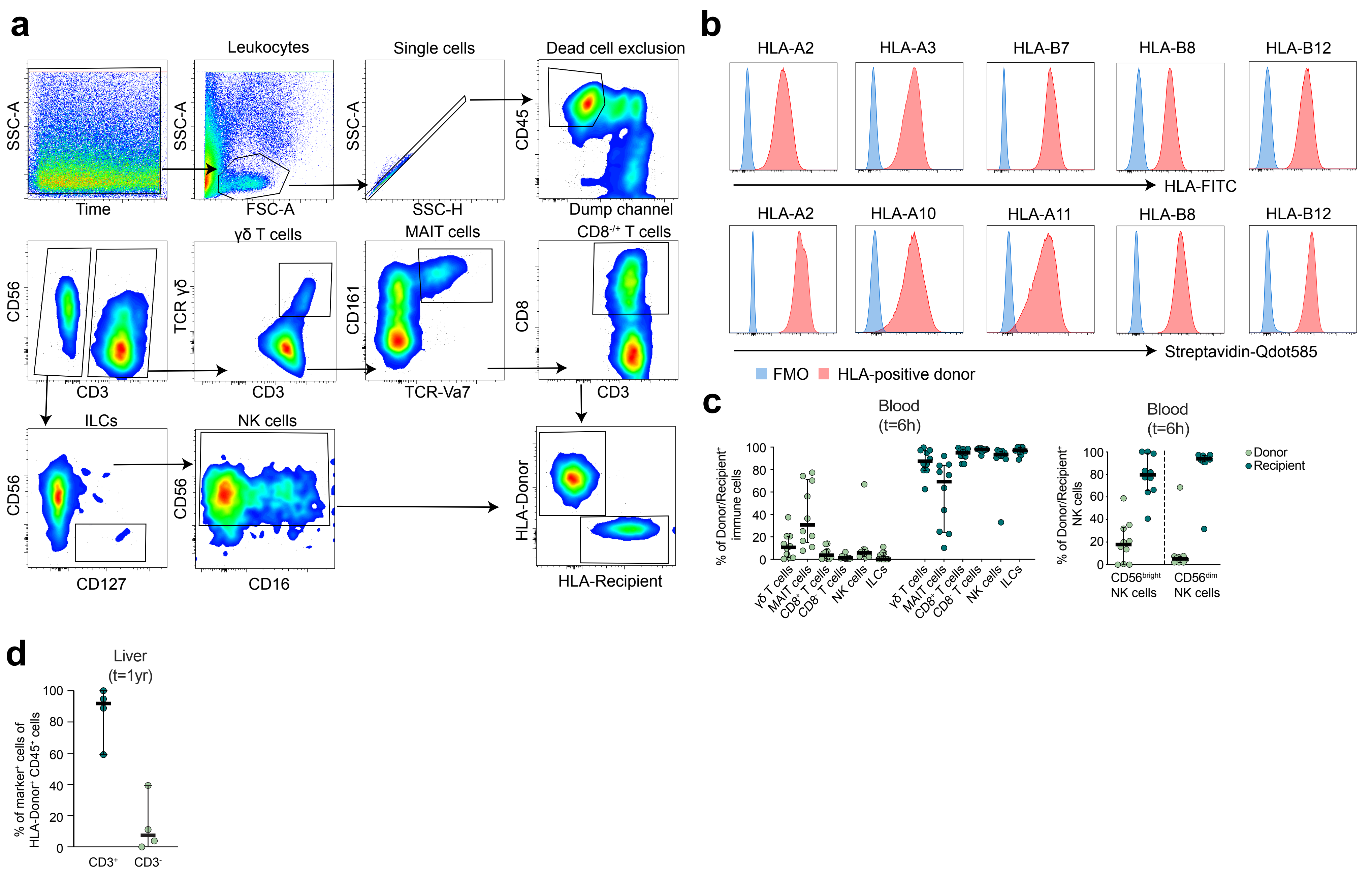

Supplementary Figure 3

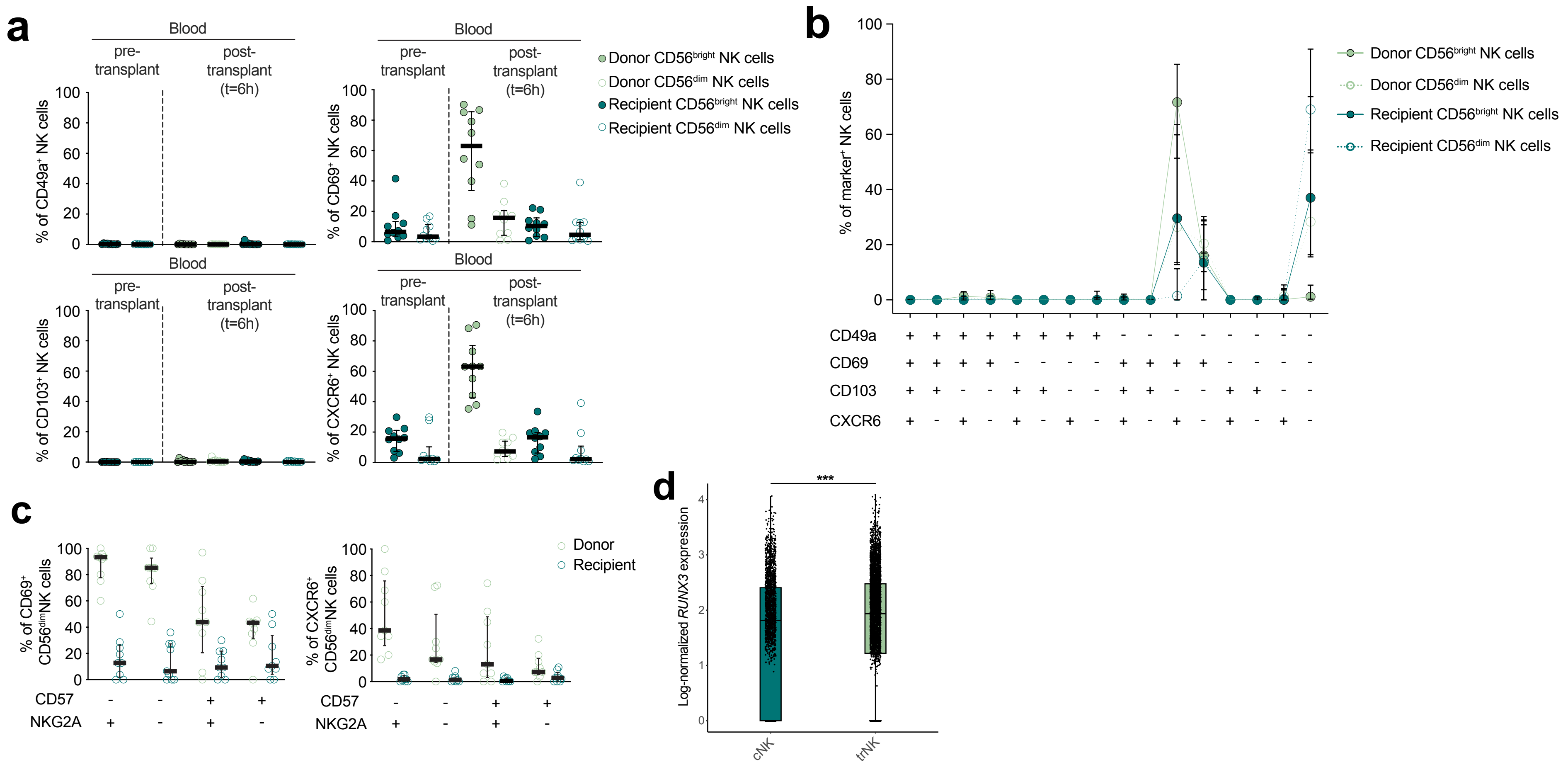

Supplementary Figure 4

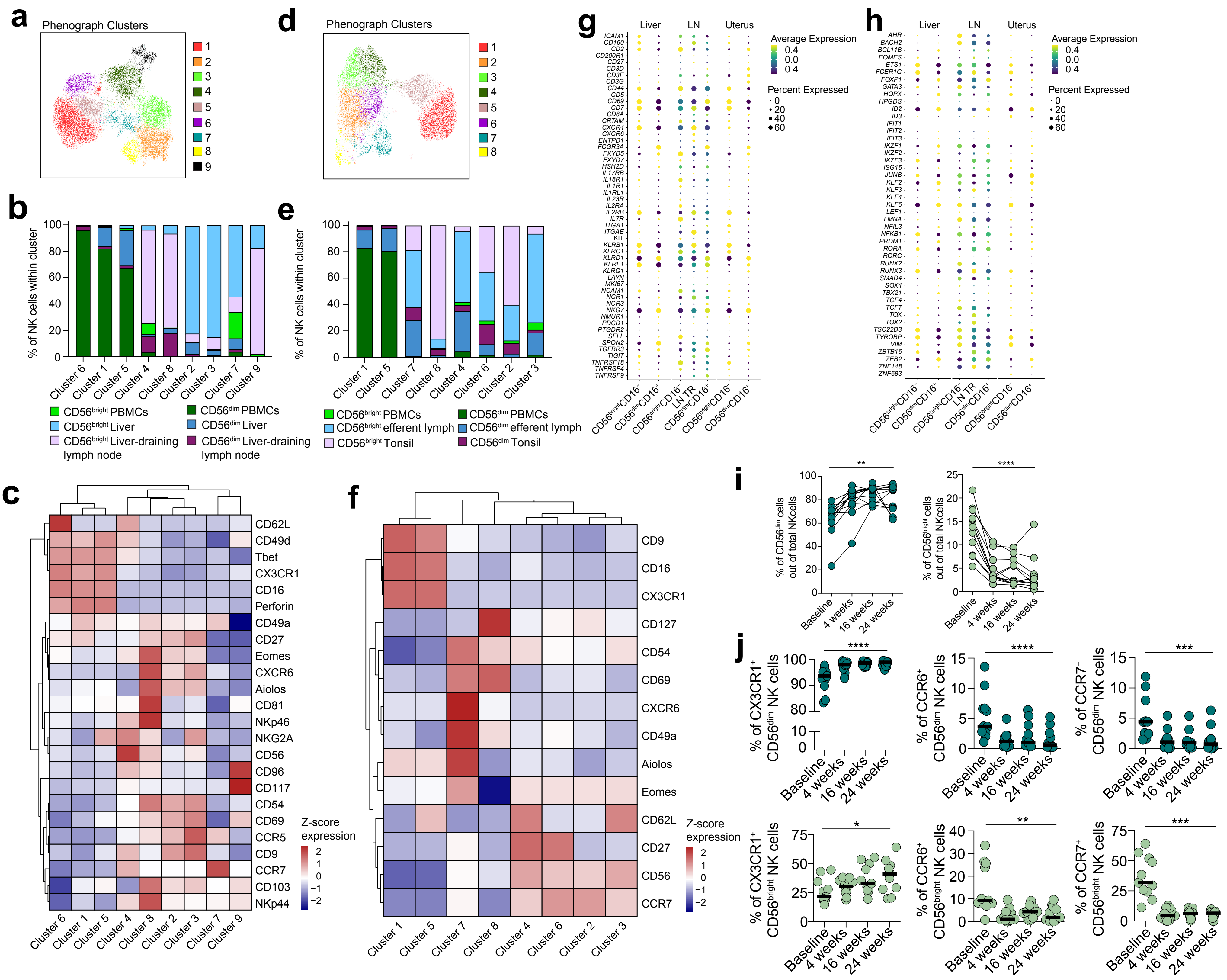

Supplementary Figure 5

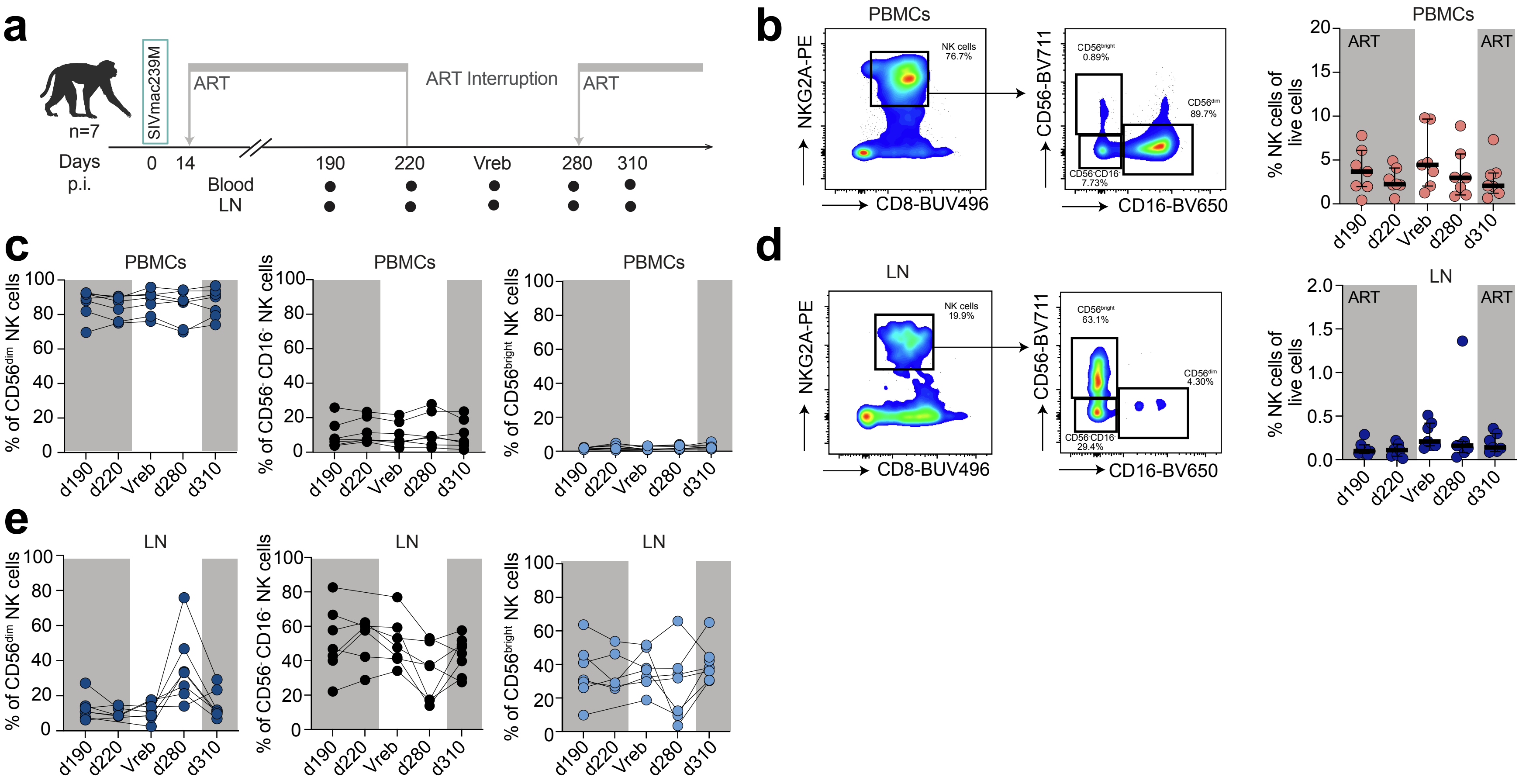

Supplementary Information

Supplementary Figure 1: $CD56^{bright}$ NK cells exhibit tissue-specific phenotypic traits. (**a**) General gating strategy for human, humanized mice and non-human primate NK cells analyzed in the present study by high dimensional flow cytometry. (**b**) Representative flow cytometry plots for definition of NK cell subpopulations and expression of CD69, granzyme B, and perforin within total NK cells in peripheral blood and tissue, respectively. (**c**) $CD56^{bright}$ NK cells from various organs of 3-6 donors were downsampled to 300-500 events per individual, concatenated (1000-3000 events in total per organ), and visualized by dimensionality reduction. UMAPs of total $CD56^{bright}$ NK cells separated in cells from diverse human tissues and matched UMAPs showing mean expression of indicated surface molecules are shown. vAT: visceral adipose tissue, sAT: subcutaneous adipose tissue. Illustrations were provided by Servier Medical Art and BioRender.

Supplementary Figure 2: Identification of donor and recipient cells using flow cytometry **(a)** General gating strategy to identify different HLA-donor^{+}/HLA-recipient^{+} immune cell populations by high dimensional flow cytometry. (**b**) Representative histograms of staining of individual samples with different specific HLA-A/B antibodies. FMO controls (blue filled histograms) and single-stain HLA molecules (red filled histograms) conjugated to FITC (upper panel) or biotin followed by Streptavidin-Qdot585 staining (lower panel) are shown. (**c**) Percentage of HLA-Donor^{+}/HLA-Recipient^{+} immune cell subsets (left panel) and $CD56^{bright/dim}$ NK cells (right panel) was determined in peripheral blood six hours after transplantation. Donor^{+} cells are depicted in light green. Recipient^{+} are depicted in dark green. Each dot represents one individual donor (n=10). Line indicates the median. Error bars display interquartile range. (**d**) Percentage of $CD3^{+}$ (dark green) and $CD3^{-}$ (light green) cells of HLA-Donor^{+} $CD45^{+}$ liver cells one year after transplantation are depicted. Each dot represents one individual donor (n=4). Line indicates the median and bars indicate interquartile range.

Supplementary Figure 3: Recipient NK cells display TR marker expression (**a**) Percentage of $CD49a^{+}$ (upper left), $CD69^{+}$ (upper right), $CD103^{+}$ (lower left) and $CXCR6^{+}$ (lower right) positive NK cells measured in peripheral blood pre-transplant and post-transplant (t=6h). Percentages are depicted for HLA-Donor^{+} $CD56^{bright}$ (filled light green dots), $CD56^{dim}$ (empty light green dots), HLA-Recipient^{+} $CD56^{bright}$ (filled dark green dots) and $CD56^{dim}$ (empty dark green dots) NK cells. Each dot represents one donor (n=10). Line indicates the

median, bars indicate interquartile range. (**b**) Boolean gating was used to assess the number of liver-derived NK cells expressing different combinations of tissue-resident markers at 6h post liver transplantation. Dot represents the median value (n=10) and error bars indicate interquartile range. (**c**) Percentage of $CD69^+$ (left) and $CXCR6^+$ (right) of HLA-$Donor^+$ (light green) and HLA-$Recipient^+$ (dark green) liver $CD56^{dim}$ NK cells at 6h post liver transplant is shown based on CD57 and NKG2A expression. Each dot represents one individual donor (n=10). Line illustrates the median and error bars represent interquartile range. (d) Log-normalized *RUNX3* expression was determined in circulating (dark green) and tissue-resident (light green) liver NK cells 6h post transplantation as measured by single-cell RNA sequencing. Each dot represents one single cell. Significant differences were determined using the non-parametric paired Wilcoxon signed rank test. ***p<0.0051

Supplementary Figure 4: Lymph fluid NK cells are phenotypically different from PB NK cells

Human efferent lymph fluid, peripheral blood (PB), tonsil, and liver-draining lymph node (LN) samples were collected for the analysis of NK cells by high-dimensional flow cytometry. (**a**) NK cells from PBMCs, liver (both n=5) and liver-derived lymph node (n=3) were downsampled, concatenated and visualized by dimensionality reduction (UMAP). Phenograph analysis was used to identify the 9 tissue-specific clusters, which are overlaid onto the UMAP and highlighted. (**b**) Stacked bar graphs show the distribution of each NK cells subset from liver, liver-draining lymph node and blood within the identified phenograph clusters. (**c**) Heatmap displaying the z-score of mean fluorescence intensity of each marker for the individual phenograph clusters from liver, liver-draining lymph node and blood derived NK cells. (**d**) Expression of surface and intracellular markers was analyzed on human PB, efferent lymph fluid and tonsil NK cells using flow cytometry. NK cells from 4 (PBMCs), 5 (efferent lymph fluid), and 3 (tonsil) donors were downsampled, concatenated and visualized by UMAP. Phenograph analysis was used to identify 8 tissue-specific clusters, which are overlaid onto the UMAP and highlighted. (**e**) Stacked bar graphs show the distribution of each NK cells subset from blood, tonsil and efferent lymph fluid within the identified phenograph clusters. (**f**) Heatmap displaying the z-score of mean fluorescence intensity of each marker for the individual phenograph cluster from blood, tonsil and efferent lymph fluid derived NK cells. Publicly available single-cell sequencing data sets from lymph node (LN)[52] and uterus[53] were integrated with single-cell sequencing data from liver generated within this study using Seurat function and gene expression is displayed for cell surface (**g**) and transcription factors (**h**)

identified within distinct NK cell population from LN, uterus and liver. Colors of the bubble plot indicate the average expression (log-normalized gene expression) while size of the bubbles indicate the percentage of cells expressing the indicated genes. (**i**) PB samples from MS patients were collected before and after start of FTY720 treatment and percentages of CD56^{bright} and CD56^{dim} cells out of total NK cells are displayed. Each dot represents one individual (n=12), and lines connect values from the same individual. **p= 0.0011, ****p<0.0001 (**j**) Expression of the chemokine receptors CX3CR1, CCR6 and CCR7 on CD56^{bright} and CD56^{dim} NK cells is shown. Each dot represents one individual and bars indicate the median (n=12). ****p<0.0001 and *p=0.012 (CX3CR1), ****p<0.0001 and **p=0.0057 (CCR6) and ***p= 0.0009 (CD56^{dim} NK cells) and ***p=0.0002 (CD56^{bright} NK cells) (CCR7). Non-parametric Friedman test was used with Dunn's post-hoc test for multiple-group comparisons was used for statistical analysis. Only comparisons between baseline and week 24 are depicted within the figure. Two-tailed p-values < 0.05 were considered significant

Supplementary Figure 5: CD56^{dim} NK cells enter lymph node tissue during inflammatory conditions

(**a**) Schematic overview of the experimental SIV infection. Seven rhesus macaques (RM) were infected with SIVmac239M and were started on d14 post infection (p.i.) on daily subcutaneous antiretroviral therapy for 220 days. Subsequently antiretroviral therapy was interrupted for a total of 60 days and reintroduced at d280p.i.. Peripheral blood and lymph nodes (LN) were analyzed at different days post infection and at point of viral rebound (Vreb) which was different depending on the animal in a time window between d234-d245p.i.. (**b**) NK cells in peripheral blood were identified as CD8α^{+}NKG2A/C^{+} and subsequently gated based on their CD56 and CD16 expression (left panel). Frequency of NK cells from live cells is depicted throughout different days post infection (right panel). Gray areas indicate time points where animals were treated with ART. Each dot represents one animal. Line indicates the median, error bars indicate the interquartile range. (**c**) Frequency of CD56^{dim} (dark blue, left panel), CD56^{-}CD16^{-} (black, middle panel) and CD56^{bright} (light blue, right panel) NK cells in peripheral blood is depicted at different days p.i.. Gray areas indicate time points where animals were treated with ART. Each dot represents one animal. Line connects matched time points from the same animal. (**d**) NK cells in lymph node (LN) were identified as CD8α^{+}NKG2A/C^{+} and subsequently gated based on their CD56 and CD16 expression (left panel). Frequency of LN NK cells is depicted throughout different days post infection (right panel). Gray areas

indicate time points where animals were treated with ART. Each dot represents one animal. Line indicates the median, error bars indicate the interquartile range. (**e**) Frequency of CD56^{dim} (dark blue, left panel), CD56^{-}CD16^{-} (black, middle panel) and CD56^{bright} (light blue, right panel) NK cells in lymph node is depicted at different days p.i.. Gray areas indicate time points where animals were treated with ART. Each dot represents one animal. Line connects matched time points from the same animal. Illustrations were provided by BioRender.

Supplementary Table 1: Typing results for HLA mismatched donor/receptor pairs

Donor		Recipient	
**HLA-A**	**HLA-B**	**HLA-A**	**HLA-B**
*02/*26	*44/*45	*03/*03	*07/*035
*03/*32	*B7/*B40	*01/*32	*08/*52
*02/*02	*B13/*B62	*03/*09	*07/*40
*02/*24	*35/*60	*01/*31	*08/*27
*01/*09	*18/*12	*03/*34	*14/*49
*02/*25	*08/*40	*02/*26	*40/40
*03/*19	*35/*47	*02/02	*15/*57
*02/*03	*35/*40	*01/*01	*08/*08
*02/*09	*15/*40	*01/*31	*08/*27
*01/*02	*16/*40	*01/*01	*08/*08

Alleles in red highlight corresponding antibody identification in flow cytometry.

Supplementary Table 2: Antibody overview

REAGENT	SOURCE	IDENTIFIER
Anti-human a4/b7-APC, clone A4B7	Millipore Milli-Mark	N/A discontinued
Mouse anti-human CCR6-Pe-Cy7, clone 11A9	BD Biosciences	Cat#560620, RRID: AB_1727440
Mouse anti-human CCR7-BV421, clone G043H7	Biolegend	Cat#353208, RRID: AB_11203894
Mouse-anti human CCR7-PerCp-Cy5.5, clone G043H7	Biolegend	Cat#353220, RRID: AB_10916121
Mouse-anti human CCR7-APC/Cy7, clone G043H7	Biolegend	Cat# 353211, RRID:AB_109 15272
Rat-anti human CCR7-BB700, clone 3D12	BD Biosciences	Cat# 566437, RRID:AB_274 4306
CD3-APC-A750, clone UCHT1	Beckman Coulter	Cat#A66329
Mouse-anti humanCD3-APC-Cy7, clone SP34-2	BD Biosciences	Cat#557757, RRID: AB_396863
Mouse anti-human CD3-BV785, clone OKT3	Biolegend	Cat#317330, RRID: AB_2563507
Mouse anti-human CD3-ECD, clone UCHT1	Beckman Coulter	Cat#IM2705U
Mouse anti-human CD3-PE-Cy5, clone UCHT1	Biolegend	Cat#300410, RRID: AB_314064
Mouse anti-human CD3-PC5.5, clone UCHT1	Beckman Coulter	Cat# A66327

Mouse anti-human CD3-BUV395, clone SP34-2	BD Biosciences	Cat#565983, RRID: AB_2739435
Mouse anti-human CD3-BUV805, clone UCHT1	BD Biosciences	Cat# 612895, RRID:AB_2870183
Mouse anti-human CD3-BV750, clone SK7	Biolegend	Cat#344845, RRID: AB_2734352
Mouse anti-human CD4-BB515, clone RPA-T4	BD Biosciences	Cat#564419, RRID: AB_2744419
Mouse anti-human CD4- APC-Cy7, clone OKT4	Biolegend	BioLegend Cat# 317418, RRID:AB_571947
Mouse anti-human CD8-APC-Cy7, clone SK1	BD Biosciences	Cat#557834, RRID: AB_396892
Mouse anti-human CD8-BV570, clone RPA-T8	Biolegend	Cat#301037, RRID: AB_10933259
Mouse anti-human CD9-BV605, clone M-L13	BD Biosciences	Cat# 743048, RRID:AB_2741244
Mouse anti-human CD9-BV605, clone M-L13	BD Biosciences	Cat# 751193, RRID:AB_2875215
Mouse anti-human CD11c-BUV661, clone B-ly6	BD Biosciences	Cat#612967, RRID: AB_2870241

Mouse anti human CD14-BV510, clone M5E2	Biolegend	Cat#301842, RRID: AB_2561946
Mouse anti-human CD14-V500, clone M5E2	BD Biosciences	Cat#561391, RRID: AB_10611856
Mouse anti-human CD14-BUV395, clone MØP9	BD Biosciences	Cat#563561, RRID: AB_2744288
Mouse anti-human CD15-V500, clone HI98	BD Biosciences	Cat#561585, RRID: AB_10896278
Mouse anti-human CD16-APC-A750, clone 3G8	Beckman Coulter	Cat# A66330
Mouse anti-human CD16-BV421, clone 3G8	BD Biosciences	Cat#562874 RRID: AB_2716865
Mouse anti-human CD16-BV711, clone 3G8	BD Biosciences	Cat#563127, RRID: AB_2732050
Mouse anti-human CD16-BUV496, clone 3G8	BD Biosciences	Cat#612944, RRID: AB_2870224
Mouse anti-human CD16-BV421, clone 3G8	BD Biosciences	Cat# 562874, RRID:AB_2716865
Mouse anti-human CD16-BV786, clone 3G8	BD Biosciences	Cat#563690, RRID: AB_2744299
Mouse anti-human CD16-ECD, clone 3G8	Beckman Coulter	Cat# A33098, RRID:AB_2728092

Mouse anti-human CD19-BUV395, clone SJ25C1	BD Biosciences	Cat#563549, RRID: AB_2738272
Mouse anti-human CD19-V500, clone HIB19	BD Biosciences	Cat# 561121, RRID: AB_10562391
Mouse anti-human CD19-PE-Cy5, clone HIB19	BD Biosciences	Cat#555414, RRID: AB_395814
Mouse anti-human CD19-BV510, clone SJ25C1	BD Biosciences	Cat#562947, RRID: AB_2737912
Mouse anti-human CD19-BV510, clone HIB19	Biolegend	Cat# 302242, RRID:AB_2561668
Mouse anti-human CD27-PE-Cy5, clone O323	ThermoFisher	Cat# 15-0279-42
Mouse anti-human CD27-BV750, clone O323	BD Biosciences	Cat# 751671, RRID:AB_2875657
Mouse anti-human CD27-BV785, clone O323	Biolegend	Cat# 302832, RRID:AB_2562674
Mouse anti-human CD28-Biotin, clone 28.2	BD Biosciences	Cat#555727, RRID: AB_396070
Mouse anti-human CD28-BUV737, clone 28.2	BD Biosciences	Cat# 612815, RRID:AB_2870140
Mouse anti-human CD45-AF700, clone HI30	Biolegend	Cat#304024, RRID: AB_493761

Mouse anti-human CD45-BV785, clone HI30	Biolegend	Cat#304048, RRID: AB_2563129
Mouse anti-human CD45-PE, clone HI30	Biolegend	Cat#304008, RRID: AB_314396
Mouse anti-human CD45-BUV805, clone HI30	BD Biosciences	Cat#612891, RRID: AB_2870179
Mouse anti-human CD49a-AF647, clone TS2/7	Biolegend	Cat#328310, RRID: AB_2129242
Mouse anti-human CD49a-BUV615, clone SR84	BD Biosciences	Custom Conjugate, Filipovic et al. (2019)[44]
Mouse anti-human CD49a-PE-Cy7, clone TS2/7	Biolegend	Cat# 328312, RRID:AB_2566272
Mouse anti-human CD49a-BB790, clone SR84	BD Biosciences	Cat# 746056, RRID:AB_2743439
Mouse anti-human CD49d-BUV661 clone L25	BD Biosciences	Cat# 750165, RRID:AB_2874370
Mouse anti-human CD54-PE-Cy5, clone HA58	BD Biosciences	Cat#555512, RRID: AB_395902
Mouse anti-human CD54-BV711, clone HA58	BD Biosciences	Cat# 564078, RRID:AB_2738579

Mouse anti-human CD54-BB700, clone HA58	BD Biosciences	Cat# 742221, RRID:AB_2871433
Mouse anti-human CD54-BUV495, clone HA58	BD Biosciences	Cat# 741152, RRID:AB_2916918
Mouse anti-human CD56-BV570, clone HCD56	Biolegend	Cat#318330, RRID: AB_2563837
Mouse anti-human CD56-BV711, clone B159	BD Biosciences	Cat# 740781, RRID:AB_2740444
Mouse anti-human CD56-BV750, clone HCD56	Biolegend	Cat# 318330, RRID: AB_2563837
Mouse anti-human CD56-BV786, clone NCAM16.2	BD Biosciences	Cat#564058, RRID: AB_2738569
Mouse anti-human CD56-APC-A750, clone N901	Beckman Coulter	Cat#B46024
Mouse anti-human CD56-ECD, clone N901	Beckman Coulter	Cat#B49214
Mouse anti-human CD56-PE, clone HCD56	Biolegend	Cat#318306, RRID: AB_604101
Mouse anti-human CD56-PC5, clone N901	Beckman Coulter	Cat# A07789, RRID:AB_1575976
Mouse anti-human CD56-PC5.5, clone N901	Beckman Coulter	Cat#B49189
Mouse anti-human CD56-PC7, clone N901	Beckman Coulter	Cat# A21692, RRID:AB_2892144
Mouse anti-human CD56-BUV737, clone NCAM16.2	BD Biosciences	Cat#564447, RRID: AB_2744432

Mouse anti-human CD56-BUV563, clone NCAM16.2	BD Biosciences	Cat#565704, RRID: AB_2744431
CD57-PE, clone HCD57	Biolegend	N/A (discontinued product)
Mouse anti-human CD57-PE-CF594, clone NK-1	BD Biosciences	Cat#562488, RRID: AB_2737625
Mouse anti-human CD57-BV605, clone Qa1704	Biolegend	Cat#393304, RRID: AB_2728426
Mouse anti-human CD57-APC-Vio770, clone TB03	Miltenyi Biotec	Cat#130-116-503, RRID: AB_2727577
Mouse anti-human CD61-BV650, clone VI-PL2	BD Biosciences	Cat#564172, RRID: AB_2738643
Mouse anti-human CD62L-BV711, clone SK11	BD Biosciences	Cat#565040, RRID: AB_2869642
Mouse anti-human CD62L-BV750, clone SK11	BD Biosciences	Cat# 747199, RRID:AB_2871927
Mouse anti-human CD62L-AF488, clone DREG-56	Biolegend	Cat# 304816, RRID: AB_528857
Mouse anti-human CD62L-Pe-Cy5, clone DREG-56	BD Biosciences	Cat# 555545, RRID:AB_395929
Mouse anti-human CD69-AF647, clone FN50	Biolegend	Cat#310918, RRID: AB_528871

Mouse anti-human CD69-BUV737, clone FN50	BD Biosciences	Cat#612817, RRID: AB_2870141
Mouse anti-human CD69-APC-Cy7, clone FN50	BD Biosciences	Cat#557756, RRID: AB_396862
Mouse anti-human CD69-BUV395, clone FN50	BD Biosciences	Cat#564364, RRID: AB_2738770
Mouse anti-human CD69-PE-Cy5, clone FN50	Biolegend	Cat# 310908, RRID:AB_314843
Mouse anti-human CD69-BV711, clone FN50	BD Biosciences	Cat# 563836, RRID:AB_2738443
Mouse anti-human CD81-BUV805, clone JS-81	BD Biosciences	Cat# 742034, RRID:AB_2871329
Mouse anti-human CD94-PE-Cy7, clone DX22	Biolegend	Cat#305516, RRID: AB_2632753
Mouse anti-human CD95-BUV737, clone DX2	BD Biosciences	Cat#612790, RRID: AB_2870117
Mouse anti-human/monkey CD95-BV605, clone DX2	Biolegend	Cat# 305628, RRID:AB_2563825
Mouse anti-human/monkey CD96-BV711, clone 6F9	BD Biosciences	Cat# 563174, RRID:AB_2738046
Mouse anti-human CD103-BUV395, clone Ber-ACT8	BD Biosciences	Cat#564346, RRID: AB_2738759

Mouse anti-human CD103-BV711, clone Ber-ACT8	BD Biosciences	Cat#563162, RRID: AB_2738039
Mouse anti-human CD103-BB660, clone Ber-ACT8	BD Biosciences	Custom Conjugate, Filipovic et al. (2019)[44]
Mouse anti-human CD103-BB700, clone Ber-ACT8	BD Biosciences	Cat# 745919, RRID:AB_2743345
Mouse anti-human CD103-Pe-Cy7, clone Ber-ACT8	Biolegend	Cat# 350212, RRID:AB_2561599
Mouse anti-human CD117-PE-Cy5.5, clone 104D2D1	Beckman Coulter	Cat#B96754
Mouse anti-human CD123-BV510, clone 6H6	Biolegend	Cat#306022, RRID: AB_2562068
Mouse anti-human CD127-BV711, clone A019D5	Biolegend	Cat#351328, RRID: AB_2562908
Mouse anti-human CD127-BV711, clone A019D5	Miltenyi Biotec	Cat# 130-113-413, RRID:AB_2726161
Mouse anti-human CD127-PE-Dazzle594, clone A019D5	Biolegend	Cat#351336, RRID: AB_2563637
Mouse anti-human CD127-PC7, clone R34.34	Beckman Coulter	Cat# A64618, RRID:AB_2833031

Mouse anti-human CD127-BUV480, clone HIL-7R-M21	BD Biosciences	Cat# 566101, RRID:AB_2869742
Mouse anti-human CD160-PE	R&D Systems	Cat#FAB6700P
Mouse anti-human CD161-BV650, clone Dx12	BD Biosciences	Cat#563864, RRID: AB_2738456
Mouse anti-human CD161-BV605, clone HP-3G10	Biolegend	Cat#339916, RRID: AB_2563607
Mouse anti-human CCR5-BUV395, clone 2D7	BD Biosciences	Cat#565224, RRID: AB_2739120
Mouse anti-human CCR5-BUV661, clone 3A9	BD Biosciences	Cat# 750299, RRID:AB_2874490
Mouse anti-human CCR5-PE, clone 3A9	BD Biosciences	Cat# 560932, RRID:AB_2033947
Mouse anti-human CXCR3-Pe-Cy5, clone 1C6	BD Biosciences	Cat#551128, RRID: AB_394061
Mouse anti-human CXCR5-Pe-Cy5, clone MU5UBEE	eBioscience	Cat# 15-9185-42, RRID:AB_2815073
Mouse anti-human CXCR6-BV421, clone K041E5	Biolegend	Cat#356014, RRID: AB_2563873
Mouse anti-human CXCR6-PE, clone K041E5	Biolegend	Cat# 356003, RRID:AB_2561739

Mouse anti-human CXCR6-BB630, clone 13B1E5	BD Biosciences	Custom Conjugate
Rat anti-human CX3CR1-FITC, clone 2A9-1	Biolegend	Cat#341606, RRID: AB_1626272
Rat anti-human CX3CR1-PE, clone 2A9-1	BD Biosciences	Cat#565796, RRID: AB_2739360
Rat anti-human CX3CR1-BV650, clone 2A9-1	Biolegend	Cat# 341625, RRID:AB_2716244
Mouse anti-human CX3CR1-PE, clone K0124E1	Biolegend	Cat# 355704, RRID:AB_2561681
Mouse anti-human DNAM-1-BV711, clone DX11	BD Biosciences	Cat#564796, RRID: AB_2738956
Mouse anti-human Aiolos-PE, clone S50-895	BD Biosciences	Cat# 564811, RRID:AB_2738966
Mouse anti-human Aiolos-PE-CF594, clone S50-895	BD Biosciences	Cat# 567871, RRID:AB_2916774
Mouse anti-human Eomes-eFluor 660, clone WD1928	eBiosciences	Cat# 50-4877-42, RRID:AB_2574229
Mouse anti-human Eomes-Pe-Cy5.5, clone WD1928	Invitrogen	Cat# 35-4877-42, RRID:AB_2848321

Mouse anti-human Eomes-Pe-Cy7, clone WD1928	eBioscience	Cat# 25-4877-42, RRID:AB_2573456
FcRy-AF700, rabbit-anti-human	EMD Milipore Mili-Mark	Conjugated in house
Mouse anti-human Granzyme B-Pe-CF594, clone GB11	BD Biosciences	Cat#561142, RRID: AB_10561690
Mouse anti-human Granzyme B-Pe-TexasRed, clone GB11	Invitrogen	Cat# GRB17, RRID:AB_1500187
Mouse anti-human Granzyme B-BB790	BD Biosciences	Custom Conjugate, Filipovic et al. (2019)[44]
Mouse anti-human HLA-A2-FITC, clone BB7.2	BD Biosciences	Cat#343304, RRID: AB_1659245
Recombinant anti-human HLA-A2-Biotin, clone REA517	Miltenyi Biotec	Cat#130-123-242, RRID: AB_2811481
Recombinant anti-human HLA-A3-FITC, clone REA950	Miltenyi Biotec	Cat#130-115-793, RRID: AB_2727190
Mouse anti-human HLA-A25/26(A10)-Biotin	OneLambda	Cat# BIH0048
Mouse anti-human HLA-A11-Biotin	OneLambda	Cat#BIH0084
Mouse anti-human HLA-B7-FITC, clone BB7.1	Invitrogen	Cat# MA1-82180, RRID:AB_931644

Recombinant anti-human HLA-B8-Biotin, clone REA145	Miltenyi Biotec	Cat#130-099-589, RRID: AB_2652007
Recombinant anti-human HLA-B8-FITC, clone REA145	Miltenyi Biotec	Cat#130-118-366, RRID: AB_2733668
Mouse anti-human HLA-B12-FITC	OneLambda	Cat#FH0066
Recombinant anti-human HLA-B12-Biotin	Miltenyi Biotech	Cat#130-099-856, RRID: AB_2652110
KIR2DL1-APC-Vio770, clone REA284	Miltenyi Biotech	Cat#130-118-345, RRID: AB_2751487
Mouse anti-human KIR2DL1/S1-PC5.5, clone EB6B	Beckman Coulter	Cat# A66898, RRID:AB_2857330
Mouse anti-human KIR2DL2/L3/S2-PC5.5, clone GL183	Beckman Coulter	Cat# A66900, RRID:AB_2857331
KIR3DL1- PerCP-Cy5.5, clone DX9	BD Pharmingen	N/A (discontinued product)
Mouse anti-human KIR3DL1/S1-PE, clone Z27.3.7	Beckman Coulter	Cat# IM3292, RRID:AB_131339
Mouse anti-human Ki-67-AF700, clone B56	BD Biosciences	Cat#561277, RRID: AB_10611571
Mouse anti-human Ki-67-BV480, clone B56	BD Biosciences	Cat# 566109, RRID:AB_2739511

Mouse anti-human Ki-67-RB780, clone B56	BD Biosciences	Cat# 568761, RRID:AB_3684525
Mouse anti-human KLRG1-BV605, clone 2F1	Biolegend	Cat# 138419, RRID:AB_2563357
Mouse anti-human NKG2A-AF488, clone 131411	R&D Systems	Cat# FAB1059G-100
Mouse anti-human NKG2A-AF700, clone 131411	R&D Systems	Cat# FAB1059N-100
Mouse anti-human NKG2A-APC, clone Z199	Beckman Coulter	Cat# A60797, RRID:AB_10643105
Recombinant anti-human NKG2A- VioBright FITC, clone REA110	Miltenyi Biotec	Cat#130-113-568, RRID: AB_2726173
Recombinant anti-human NKG2A-PE, clone REA110	Miltenyi Biotec	Cat#130-113-566, RRID: AB_2726171
Mouse anti-human NKG2A-PE, clone Z199	Beckman Coulter	Cat# IM3291U, RRID:AB_10643228
Mouse anti-human NKG2A-Pe-Cy7, clone Z199	Beckman Coulter	Cat# B10246, RRID:AB_2687887
Mouse anti-human NKG2A-BB700, clone 131411	BD Biosciences	Cat#747926, RRID: AB_2872387
Recombinant anti-human NKG2C- VioBright FITC, clone REA205	Miltenyi Biotec	Cat#130-117-707, RRID: AB_2728023

Mouse anti-human NKG2D-PE-Cy7, clone 1D11	Biolegend	Cat#320812, RRID: AB_2234394
Mouse anti-human NKp30-BV605, clone p30-15	BD Biosciences	Cat#563384, RRID: AB_2738170
Mouse anti-human NKp44-BUV737, clone p44-8	BD Biosciences	Cat# 744301, RRID:AB_2742131
Mouse anti-human NKp44-BUV737, clone p44-8	BD Biosciences	Cat# 749172, RRID:AB_2873552
Mouse anti-human NKp46-BV786, clone 9E2/NKp46	BD Biosciences	Cat#563329, RRID: AB_2738139
Mouse anti-human NKp46-BV650, clone 9E2	Biolegend	Cat# 331927, RRID:AB_2562442
Mouse anti-human NKp80-APC, clone 5D12	Biolegend	Cat#346708, RRID: AB_2044041
Mouse anti-human PD-1-BV421, clone EH12.2H7	Biolegend	Cat#329920, RRID: AB_10960742
Mouse anti-human PD-1-BV785, clone EH12.2H7	Biolegend	Cat# 329930, RRID:AB_2563443
Mouse anti-human Perforin-BV421, clone dG9	Biolegend	Cat#308122, RRID: AB_2566204
Mouse anti-human Perforin-BV421, clone B-D48	Biolegegend	Cat# 353307, RRID:AB_11149688

Mouse anti-human Perforin-PE-Cy7, clone B-D48	Biolegend	Cat#353316, RRID: AB_2571973
Mouse anti-human Perforin-BB755, clone deltaG9	BD Biosciences	Custom Conjugate, Filipovic et al. (2019)[44]
Mouse anti-human Runx3-PE, clone R3-5G4	BD Biosciences	Cat# 564814, RRID:AB_2738969
Mouse anti-human Sialyl Lewis X-BV711, clone CSLEX1	BD Biosciences	Cat#563910, RRID: AB_2738481
Recombinant anti-human Siglec-7-PercP-Vio 700, clone REA214	Miltenyi Biotec	Cat#130-100-979, RRID: AB_2657543
Mouse anti-human Syk-PE, clone 4D10.1	Affymetrix eBiosciences (now Life Technologies)	Cat#15557076
Mouse anti-human T-bet-PE-Dazzle 594, clone 4B10	Biolegend	Cat#644828, RRID: AB_2565677
Mouse anti-human T-bet-BV421, clone 4B10	Biolegend	Cat# 644815, RRID:AB_10896427
Mouse anti-human TIGIT-PE-Cy7, clone 1G9	Biolegend	Cat#142108, RRID: AB_2565648
Mouse anti-human Tim-3-BV650, clone F38-2E2	Biolegend	Cat#345028, RRID: AB_2565829

Mouse anti-human TCR PAN y/d-PC5.5, clone IMMU510	Beckman Coulter	Cat# A99021, RRID:AB_2910257
Mouse anti-human TCRVa7.2-PE-Cy7, clone 3C10	Biolegend	Cat#351712, RRID: AB_2561994
Rat anti-mouse CD45-AF700, clone 30F11	Biolegend	Cat#103128, RRID: AB_493715
Mouse anti-human/monkey CD3-AP-Cy7, clone SP34-2	BD Biosciences	Cat#557757, RRID: AB_396863
Mouse anti-human/monkey CD3-BUV395, clone SP34-2	BD Biosciences	Cat# 564117, RRID:AB_2738603
Mouse anti-human CD3-BV570, clone UCHT1	Biolegend	Cat# 300436, RRID:AB_2562124
Mouse anti-human/monkey CD8a-BV570, clone RPA-T8	Biolegend	Cat#301038, RRID: AB_2563213
Mouse anti-human/monkey CD8a-BUV496, clone RPA-T8	BD Biosciences	Cat# 612942, RRID:AB_2870223
Mouse anti-human/monkey CD14-APC-Cy7, clone M5E2	Biolegend	Cat#301820, RRID: AB_493695
Mouse anti-human/monkey CD14-BV510, clone M5E2	Biolegend	Cat# 301842, RRID:AB_2561946
Mouse anti-human/monkey CD16-BV650, clone 3G8	Biolegend	Cat#302042, RRID: AB_2563801

Mouse anti-human/monkey CD16-BUV496, clone 3G8	BD Biosciences	Cat# 564653, RRID:AB_2744294
Mouse anti-human/monkey CD20-APC-Cy7, clone 2H7	Biolegend	Cat#302314, RRID: AB_314262
Mouse anti-human/monkey CD20-PerCP/Cy5.5, clone 2H7	Biolegend	Cat# 302326, RRID:AB_893283
Mouse anti-human/monkey CD56-PE-Cy7, clone B159	BD Biosciences	Cat#557747, RRID: AB_396853
Mouse anti-human Granzyme B-PE Texas Red, clone GB11	Invitrogen	Cat# GRB17, RRID:AB_2536540
Mouse anti-human Granzyme B-AF647, clone GB11	BD Biosciences	Cat# 560212, RRID:AB_11154033
Mouse anti-human/monkey HLA-DR-BV421, clone G46-6	BD Biosciences	Cat#562804, RRID: AB_2687421
Mouse anti-human/monkey HLA-DR-BV605, clone G46-6	BD Biosciences	Cat#562845
Mouse anti-human/monkey HLA-DR-BV650, clone L243	Biolegend	Cat# 307650, RRID:AB_2563828
Mouse anti-human/monkey HLA-DR-BUV615, clone G46-6	BD Biosciences	Cat# 751142, RRID:AB_2875168
Mouse anti-human/non-human primates Perforin-FITC, clone Pf-344	Mabtech AB	Cat#3465-7

Streptavidin-BB630	BD Biosciences	Costum Conjugate, Filipovic et al. (2019)[44]
Streptavidin-QD585	Life Technologies	Cat#Q10111MP
