# Supplementary material for: Transient tissue residency and lymphatic egress define human CD56bright NK cell homeostasis
Source: Nat Immunol. 2025 Oct 14;26(11):2004–15. doi: 10.1038/s41590-025-02290-9 (PMC12571907; doi:10.1038/s41590-025-02290-9)
Supplement: Supplementary file 2 — Reporting Summary [file 41590_2025_2290_MOESM2_ESM.pdf]

nature portfolio

Corresponding author(s): Niklas K Björkström

Last updated by author(s): Aug 14, 2025

Reporting Summary

Nature Portfolio wishes to improve the reproducibility of the work that we publish. This form provides structure for consistency and transparency in reporting. For further information on Nature Portfolio policies, see our Editorial Policies and the Editorial Policy Checklist.

Statistics

For all statistical analyses, confirm that the following items are present in the figure legend, table legend, main text, or Methods section.

n/a	Confirmed	
☐	☒	The exact sample size (*n*) for each experimental group/condition, given as a discrete number and unit of measurement
☐	☒	A statement on whether measurements were taken from distinct samples or whether the same sample was measured repeatedly
☐	☒	The statistical test(s) used AND whether they are one- or two-sided<br>*Only common tests should be described solely by name; describe more complex techniques in the Methods section.*
☐	☒	A description of all covariates tested
☐	☒	A description of any assumptions or corrections, such as tests of normality and adjustment for multiple comparisons
☐	☒	A full description of the statistical parameters including central tendency (e.g. means) or other basic estimates (e.g. regression coefficient) AND variation (e.g. standard deviation) or associated estimates of uncertainty (e.g. confidence intervals)
☐	☒	For null hypothesis testing, the test statistic (e.g. *F*, *t*, *r*) with confidence intervals, effect sizes, degrees of freedom and *P* value noted<br>*Give P values as exact values whenever suitable.*
☒	☐	For Bayesian analysis, information on the choice of priors and Markov chain Monte Carlo settings
☒	☐	For hierarchical and complex designs, identification of the appropriate level for tests and full reporting of outcomes
☒	☐	Estimates of effect sizes (e.g. Cohen's *d*, Pearson's *r*), indicating how they were calculated

Our web collection on statistics for biologists contains articles on many of the points above.

Software and code

Policy information about availability of computer code

Data collection	Flow cytometry data was collected on a BD LSR Fortessa, BD LSRII, BD FACSymphony A5 (all BD Biosciences) or Navios flow cytometer (Beckman Coulter).<br>scRNA sequencing was performed using a DNBSEQ platform (MGI Tech).
Data analysis	Standard flow cytometry data analysis was performed using FlowJo version 10.4.2. or Kaluza software 1.5a (Beckman Coulter, Brea, CA, USA). Analysis and visualization of surface marker expression on NK cell subsets was further performed in R Studio (v1.3.959) using the packages ggplot2 (v3.3.2), reshape2 (v1.4.4), tidyr (v1.1.1), viridis (v0.5.1), dplyr (v1.0.1) and pheatmap(v1.0v12).<br><br>scRNAseq analysis was performed in R Studio (v4.2.1). FASTQ files from were demultiplexed using deML (v1.1.3), samples were quality-filtered, aligned to the hg38 reference genome, and quantified based on GENCODE (v35) gene annotations using the zUMIs pipeline (v2.9.4f). CellBender (v0.1.058) was employed to remove empty droplets and background noise, and Solo (v1.059) was used to eliminate doublets. Individual cell identities were assigned by using cellsnp-lite (v1.2.0) and cells were subsequently assigned to eight genetic identities (--nDonor 8) using Vireo (v0.4.2). Genetic identities were visualized using the ggalluvial package (v0.12.3). Expression matrices were merged, log-normalized, and the normalized expression data of the top 2000 most variable features (selection.method = 'vst') were scaled in Seurat (v4.1.062). Expression data from different samples were integrated by Harmony (v0.163). Azimuth human liver and PBMC references (https://azimuth.hubmapconsortium.org/references/) were used to annotate major cell types in Seurat. RNA velocity analysis was conducted using Velocyto (v0.6). Count matrices were then imported into scVelo. Velocity was regularized using the CellRank package (v1.5.1). Top 50 putative driver genes were plotted using the Complex Heatmap package (v.2.18.0). Annotated NK cells from two publicly available datasets as well as our generated liver biopsy single-cell sequencing dataset were merged using the merge () function in the Seurat package (v4.1.0), resulting in a combined dataset of 16,743 cells. Preprocessing was performed separately per donor, including normalization (NormalizeData()), identification of variable features (FindVariableFeatures()), regression of technical variables (nCount_RNA, pct_mt) during

scaling (ScaleData()), and principal component analysis (RunPCA()). Batch correction and integration across donors were conducted using the batchelor package's FastMNNIntegration() function via IntegrateLayers() in Seurat, and dimensionality reduction was performed with UMAP on the integrated space (RunUMAP(), method = "uwot"). Clustering was carried out using mutual nearest neighbor (MNN) graph construction (FindNeighbors()) and modularity-based clustering (FindClusters(), resolution = 0.2, method = 4). Metadata such as organ and cell type were annotated as ordered factors to facilitate downstream visualization. Cell type distributions and gene expression patterns of surface markers and transcription factors were visualized using dittoDimPlot() from the dittoSeq package (v.1.18.0) and DotPlot_scCustom() with the scCustomize (v.3.0.1) and viridis packages (v.0.6.5) for customized dot plots.

Statistical analysis was conducted using GraphPad Prism version 7 (GraphPad Software). Non-parametric Wilcoxon matched rank test was used for matched two-group comparisons. Non-parametric Kruskal-Wallis test for unmatched and Friedman test for matched samples were used in combination with the Dunn's post-hoc test for multiple-group comparisons. P-values < 0.05 were considered significant, * p < 0.05; ** p < 0.01; *** p < 0.001. Where indicated, z-score of median fluorescence intensity or patient clinical parameters (MFI) were calculated as follows: $z = ((x-\mu))/\sigma$, being x = raw score, μ = mean of sample distribution and σ = standard deviation.

For manuscripts utilizing custom algorithms or software that are central to the research but not yet described in published literature, software must be made available to editors and reviewers. We strongly encourage code deposition in a community repository (e.g. GitHub). See the Nature Portfolio guidelines for submitting code & software for further information.

Data

Policy information about availability of data

All manuscripts must include a data availability statement. This statement should provide the following information, where applicable:

- Accession codes, unique identifiers, or web links for publicly available datasets
- A description of any restrictions on data availability
- For clinical datasets or third party data, please ensure that the statement adheres to our policy

All figures have corresponding primary data which is available upon request from the corresponding author. Sequencing data have been deposited to the NIH under accession number GSE246994.

Research involving human participants, their data, or biological material

Policy information about studies with human participants or human data. See also policy information about sex, gender (identity/presentation), and sexual orientation and race, ethnicity and racism.

Reporting on sex and gender

Sex was not considered in the design of this study due to limited sample availability.

Reporting on race, ethnicity, or other socially relevant groupings

Not relevant.

Population characteristics

Peripheral blood samples: Peripheral blood samples were collected from random healthy donors. No information on sex or age was collected.

Tissue samples: Disease-unaffected duodenum tissue samples were obtained from patients with intraductal papillary mucinous neoplasm (IPMN) undergoing pancreatoduodenectomy. Liver-draining lymph nodes were collected from patients undergoing distal pancreatectomy. Liver tissue samples were obtained from organ donor livers not used for transplantation, uterine tissues were taken during hysterectomy performed for benign reasons, and tonsils were obtained from patients with obstructive sleep apnea syndrome. Afferent and efferent venous blood from human liver, liver tissue, and blood samples were taken during open abdominal surgery or wedged hepatic venous pressure measurement.

CD34+ cells: Human CD34+ cells for the generation of humanized mice were obtained from umbilical cord blood.

Liver transplant cohort: Liver biopsies and PB samples from patients undergoing liver transplantation were collected at different time points before and after transplantation.

Efferent lymph fluid: Efferent lymph fluid and peripheral blood were taken from patients with leakages of their thoracic duct, lymphatic disorders, or trauma to the duct.

FTY720 treatment cohort: Peripheral blood samples were taken from patients with relapsing-remitting multiple sclerosis.

Recruitment

Blood, tissue and lymph donors were recruited at Karolinska University Hospital, Stockholm, at the Hannover Medical School, Hannover, at the University of Pennsylvania and Children's Hospital of Philadelphia and at the Department of Neurology, University of Muenster, Muenster. Due to the collection at multiple clinical sites and trough clinical partners, no self-selection bias was introduced that could impact the results. Written informed consent was obtained from all patients described above.

Ethics oversight

The studies were approved by the Regional Ethics Committee, Stockholm County, Stockholm, Sweden, Hannover Medical School, the German competent authority (Federal Institute for Drugs and Medical Devices), and the institutional review board of the University of Pennsylvania, Children's Hospital of Philadelphia, and performed in accordance with the Declaration of Helsinki.

Note that full information on the approval of the study protocol must also be provided in the manuscript.

Field-specific reporting

Please select the one below that is the best fit for your research. If you are not sure, read the appropriate sections before making your selection.

☒ Life sciences ☐ Behavioural & social sciences ☐ Ecological, evolutionary & environmental sciences

For a reference copy of the document with all sections, see nature.com/documents/nr-reporting-summary-flat.pdf

Life sciences study design

All studies must disclose on these points even when the disclosure is negative.

Sample size	Sample sizes were determined based on resources and availability. No statistical methods were used to predetermine sample size number.
Data exclusions	Low quality cells that did not fulfill the quality control criteria from the scRNAseq were excluded from subsequent analysis.<br>Figure 1b and e: CD56bright NK cells from duodenum samples were excluded from subsequent gating and analysis (%of CD69, %of Perforin and %GrzB) due to low cell frequencies in cell population that prevented conclusive gating and analysis. Lack of sample is marked with n.a. in respective figures.<br>Supplementary Figure 5: NK cell subpopulations (CD56bright/CD56dim) were not included if there were less than 15 events in the final population.
Replication	We used several approaches to ensure reproducibility. For each conclusion multiple biological replicates were included to ensure reproducible results. In addition, we used complementary experimental animal models in parallel with human sample material to verify reproducibility of the observed results.
Randomization	Participants were allocated according to sample collection scheme without randomization. Animal experiments did not include different experimental groups and were therefore not randomized.
Blinding	Blinding was not performed for the acquisition and analysis of the data. Experimental observations would be consistent irrespective of blinding. Conclusions drawn in this manuscript were based on multiple donors and distinct experimental models to confirm observations.

Reporting for specific materials, systems and methods

We require information from authors about some types of materials, experimental systems and methods used in many studies. Here, indicate whether each material, system or method listed is relevant to your study. If you are not sure if a list item applies to your research, read the appropriate section before selecting a response.

Materials & experimental systems

n/a	Involved in the study	
☐	☒	Antibodies
☒	☐	Eukaryotic cell lines
☒	☐	Palaeontology and archaeology
☐	☒	Animals and other organisms
☒	☐	Clinical data
☒	☐	Dual use research of concern
☒	☐	Plants

Methods

n/a	Involved in the study	
☒	☐	ChIP-seq
☐	☒	Flow cytometry
☒	☐	MRI-based neuroimaging

Antibodies

Antibodies used

Anti-human a4/b7-APC, clone A4B7 Millipore Milli-Mark N/A discontinued
Mouse anti-human CCR6-Pe-Cy7, clone 11A9 BD Biosciences Cat#560620, RRID: AB_1727440
Mouse anti-human CCR7-BV421, clone G043H7 Biolegend Cat#353208, RRID: AB_11203894
Mouse-anti human CCR7-PerCp-Cy5.5, clone G043H7 Biolegend Cat#353220, RRID: AB_10916121
Mouse-anti human CCR7-APC/Cy7, clone G043H7 Biolegend Cat# 353211, RRID:AB_10915272
Rat-anti human CCR7-BB700, clone 3D12 BD Biosciences Cat# 566437, RRID:AB_2744306
CD3-APC-A750, clone UCHT1 Beckman Coulter Cat#A66329
Mouse-anti humanCD3-APC-Cy7, clone SP34-2 BD Biosciences Cat#557757, RRID: AB_396863
Mouse anti-human CD3-BV785, clone OKT3 Biolegend Cat#317330, RRID: AB_2563507
Mouse anti-human CD3-ECD, clone UCHT1 Beckman Coulter Cat#IM2705U
Mouse anti-human CD3-PE-Cy5, clone UCHT1 Biolegend Cat#300410, RRID: AB_314064
Mouse anti-human CD3-PC5.5, clone UCHT1 Beckman Coulter Cat# A66327
Mouse anti-human CD3-BUV395, clone SP34-2 BD Biosciences Cat#565983, RRID: AB_2739435
Mouse anti-human CD3-BUV805, clone UCHT1 BD Biosciences Cat# 612895, RRID:AB_2870183
Mouse anti-human CD3-BV750, clone SK7 Biolegend Cat#344845, RRID: AB_2734352
Mouse anti-human CD4-BB515, clone RPA-T4 BD Biosciences Cat#564419, RRID: AB_2744419
Mouse anti-human CD4- APC-Cy7, clone OKT4 Biolegend BioLegend Cat# 317418, RRID:AB_571947

Mouse anti-human CD8-APC-Cy7, clone SK1 BD Biosciences Cat#557834, RRID: AB_396892
Mouse anti-human CD8-BV570, clone RPA-T8 Biolegend Cat#301037, RRID: AB_10933259
Mouse anti-human CD9-BV605, clone M-L13 BD Biosciences Cat# 743048, RRID:AB_2741244
Mouse anti-human CD9-BV605, clone M-L13 BD Biosciences Cat# 751193, RRID:AB_2875215
Mouse anti-human CD11c-BUV661, clone B-ly6 BD Biosciences Cat#612967, RRID: AB_2870241
Mouse anti human CD14-BV510, clone M5E2 Biolegend Cat#301842, RRID: AB_2561946
Mouse anti-human CD14-V500, clone M5E2 BD Biosciences Cat#561391, RRID: AB_10611856
Mouse anti-human CD14-BUV395, clone MØP9 BD Biosciences Cat#563561, RRID: AB_2744288
Mouse anti-human CD15-V500, clone HI98 BD Biosciences Cat#561585, RRID: AB_10896278
Mouse anti-human CD16-APC-A750, clone 3G8 Beckman Coulter Cat# A66330
Mouse anti-human CD16-BV421, clone 3G8 BD Biosciences Cat#562874 RRID: AB_2716865
Mouse anti-human CD16-BV711, clone 3G8 BD Biosciences Cat#563127, RRID: AB_2732050
Mouse anti-human CD16-BUV496, clone 3G8 BD Biosciences Cat#612944, RRID: AB_2870224
Mouse anti-human CD16-BV421, clone 3G8 BD Biosciences Cat# 562874, RRID:AB_2716865
Mouse anti-human CD16-BV786, clone 3G8 BD Biosciences Cat#563690, RRID: AB_2744299
Mouse anti-human CD16-ECD, clone 3G8 Beckman Coulter Cat# A33098, RRID:AB_2728092
Mouse anti-human CD19-BUV395, clone SJ25C1 BD Biosciences Cat#563549, RRID: AB_2738272
Mouse anti-human CD19-V500, clone HIB19 BD Biosciences Cat# 561121, RRID: AB_10562391
Mouse anti-human CD19-PE-Cy5, clone HIB19 BD Biosciences Cat#555414, RRID: AB_395814
Mouse anti-human CD19-BV510, clone SJ25C1 BD Biosciences Cat#562947, RRID: AB_2737912
Mouse anti-human CD19-BV510, clone HIB19 Biolegend Cat# 302242, RRID:AB_2561668
Mouse anti-human CD27-PE-Cy5, clone O323 ThermoFisher Cat# 15-0279-42
Mouse anti-human CD27-BV750, clone O323 BD Biosciences Cat# 751671, RRID:AB_2875657
Mouse anti-human CD27-BV785, clone O323 Biolegend Cat# 302832, RRID:AB_2562674
Mouse anti-human CD28-Biotin, clone 28.2 BD Biosciences Cat#555727, RRID: AB_396070
Mouse anti-human CD28-BUV737, clone 28.2 BD Biosciences Cat# 612815, RRID:AB_2870140
Mouse anti-human CD45-AF700, clone HI30 Biolegend Cat#304024, RRID: AB_493761
Mouse anti-human CD45-BV785, clone HI30 Biolegend Cat#304048, RRID: AB_2563129
Mouse anti-human CD45-PE, clone HI30 Biolegend Cat#304008, RRID: AB_314396
Mouse anti-human CD45-BUV805, clone HI30 BD Biosciences Cat#612891, RRID: AB_2870179
Mouse anti-human CD49a-AF647, clone TS2/7 Biolegend Cat#328310, RRID: AB_2129242
Mouse anti-human CD49a-BUV615, clone SR84 BD Biosciences Custom Conjugate, Filipovic et al. (2019)44
Mouse anti-human CD49a-PE-Cy7, clone TS2/7 Biolegend Cat# 328312, RRID:AB_2566272
Mouse anti-human CD49a-BB790, clone SR84 BD Biosciences Cat# 746056, RRID:AB_2743439
Mouse anti-human CD49d-BUV661 clone L25 BD Biosciences Cat# 750165, RRID:AB_2874370
Mouse anti-human CD54-PE-Cy5, clone HA58
BD Biosciences Cat#555512, RRID: AB_395902
Mouse anti-human CD54-BV711, clone HA58
BD Biosciences Cat# 564078, RRID:AB_2738579
Mouse anti-human CD54-BB700, clone HA58
BD Biosciences Cat# 742221, RRID:AB_2871433
Mouse anti-human CD54-BUV495, clone HA58
BD Biosciences Cat# 741152, RRID:AB_2916918
Mouse anti-human CD56-BV570, clone HCD56 Biolegend Cat#318330, RRID: AB_2563837
Mouse anti-human CD56-BV711, clone B159 BD Biosciences Cat# 740781, RRID:AB_2740444
Mouse anti-human CD56-BV750, clone HCD56 Biolegend Cat# 318330, RRID: AB_2563837
Mouse anti-human CD56-BV786, clone NCAM16.2 BD Biosciences Cat#564058, RRID: AB_2738569
Mouse anti-human CD56-APC-A750, clone N901 Beckman Coulter Cat#B46024
Mouse anti-human CD56-ECD, clone N901 Beckman Coulter Cat#B49214
Mouse anti-human CD56-PE, clone HCD56 Biolegend Cat#318306, RRID: AB_604101
Mouse anti-human CD56-PC5, clone N901 Beckman Coulter Cat# A07789, RRID:AB_1575976
Mouse anti-human CD56-PC5.5, clone N901 Beckman Coulter Cat#B49189
Mouse anti-human CD56-PC7, clone N901 Beckman Coulter Cat# A21692, RRID:AB_2892144
Mouse anti-human CD56-BUV737, clone NCAM16.2 BD Biosciences Cat#564447, RRID: AB_2744432
Mouse anti-human CD56-BUV563, clone NCAM16.2 BD Biosciences Cat#565704, RRID: AB_2744431
CD57-PE, clone HCD57 Biolegend N/A (discontinued product)
Mouse anti-human CD57-PE-CF594, clone NK-1 BD Biosciences Cat#562488, RRID: AB_2737625
Mouse anti-human CD57-BV605, clone Qa1704 Biolegend Cat#393304, RRID: AB_2728426
Mouse anti-human CD57-APC-Vio770, clone TB03 Miltenyi Biotec Cat#130-116-503, RRID: AB_2727577
Mouse anti-human CD61-BV650, clone VI-PL2 BD Biosciences Cat#564172, RRID: AB_2738643
Mouse anti-human CD62L-BV711, clone SK11 BD Biosciences Cat#565040, RRID: AB_2869642
Mouse anti-human CD62L-BV750, clone SK11 BD Biosciences Cat# 747199, RRID:AB_2871927
Mouse anti-human CD62L-AF488, clone DREG-56 Biolegend Cat# 304816, RRID: AB_528857
Mouse anti-human CD62L-Pe-Cy5, clone DREG-56 BD Biosciences Cat# 555545, RRID:AB_395929
Mouse anti-human CD69-AF647, clone FN50 Biolegend Cat#310918, RRID: AB_528871
Mouse anti-human CD69-BUV737, clone FN50 BD Biosciences Cat#612817, RRID: AB_2870141
Mouse anti-human CD69-APC-Cy7, clone FN50 BD Biosciences Cat#557756, RRID: AB_396862
Mouse anti-human CD69-BUV395, clone FN50 BD Biosciences Cat#564364, RRID: AB_2738770
Mouse anti-human CD69-PE-Cy5, clone FN50 Biolegend Cat# 310908, RRID:AB_314843
Mouse anti-human CD69-BV711, clone FN50 BD Biosciences Cat# 563836, RRID:AB_2738443
Mouse anti-human CD81-BUV805, clone JS-81 BD Biosciences Cat# 742034, RRID:AB_2871329
Mouse anti-human CD94-PE-Cy7, clone DX22 Biolegend Cat#305516, RRID: AB_2632753
Mouse anti-human CD95-BUV737, clone DX2 BD Biosciences Cat#612790, RRID: AB_2870117
Mouse anti-human/monkey CD95-BV605, clone DX2 Biolegend Cat# 305628, RRID:AB_2563825
Mouse anti-human/monkey CD96-BV711, clone 6F9 BD Biosciences Cat# 563174, RRID:AB_2738046
Mouse anti-human CD103-BUV395, clone Ber-ACT8 BD Biosciences Cat#564346, RRID: AB_2738759

Mouse anti-human CD103-BV711, clone Ber-ACT8 BD Biosciences Cat#563162, RRID: AB_2738039
Mouse anti-human CD103-BB660, clone Ber-ACT8 BD Biosciences Custom Conjugate, Filipovic et al. (2019)44
Mouse anti-human CD103-BB700, clone Ber-ACT8 BD Biosciences Cat# 745919, RRID:AB_2743345
Mouse anti-human CD103-Pe-Cy7, clone Ber-ACT8 Biolegend Cat# 350212, RRID:AB_2561599
Mouse anti-human CD117-PE-Cy5.5, clone 104D2D1 Beckman Coulter Cat#B96754
Mouse anti-human CD123-BV510, clone 6H6 Biolegend Cat#306022, RRID: AB_2562068
Mouse anti-human CD127-BV711, clone A019D5 Biolegend Cat#351328, RRID: AB_2562908
Mouse anti-human CD127-BV711, clone A019D5 Miltenyi Biotec Cat# 130-113-413, RRID:AB_2726161
Mouse anti-human CD127-PE-Dazzle594, clone A019D5 Biolegend Cat#351336, RRID: AB_2563637
Mouse anti-human CD127-PC7, clone R34.34 Beckman Coulter Cat# A64618, RRID:AB_2833031
Mouse anti-human CD127-BUV480, clone HIL-7R-M21 BD Biosciences Cat# 566101, RRID:AB_2869742
Mouse anti-human CD160-PE R&D Systems Cat#FAB6700P
Mouse anti-human CD161-BV650, clone Dx12 BD Biosciences Cat#563864, RRID: AB_2738456
Mouse anti-human CD161-BV605, clone HP-3G10 Biolegend Cat#339916, RRID: AB_2563607
Mouse anti-human CCR5-BUV395, clone 2D7 BD Biosciences Cat#565224, RRID: AB_2739120
Mouse anti-human CCR5-BUV661, clone 3A9 BD Biosciences Cat# 750299, RRID:AB_2874490
Mouse anti-human CCR5-PE, clone 3A9 BD Biosciences Cat# 560932, RRID:AB_2033947
Mouse anti-human CXCR3-Pe-Cy5, clone 1C6 BD Biosciences Cat#551128, RRID: AB_394061
Mouse anti-human CXCR5-Pe-Cy5, clone MU5UBEE eBioscience Cat# 15-9185-42, RRID:AB_2815073
Mouse anti-human CXCR6-BV421, clone K041E5 Biolegend Cat#356014, RRID: AB_2563873
Mouse anti-human CXCR6-PE, clone K041E5 Biolegend Cat# 356003, RRID:AB_2561739
Mouse anti-human CXCR6-BB630, clone 13B1E5 BD Biosciences Custom Conjugate
Rat anti-human CX3CR1-FITC, clone 2A9-1 Biolegend Cat#341606, RRID: AB_1626272
Rat anti-human CX3CR1-PE, clone 2A9-1 BD Biosciences Cat#565796, RRID: AB_2739360
Rat anti-human CX3CR1-BV650, clone 2A9-1 Biolegend Cat# 341625, RRID:AB_2716244
Mouse anti-human CX3CR1-PE, clone K0124E1 Biolegend Cat# 355704, RRID:AB_2561681
Mouse anti-human DNAM-1-BV711, clone DX11 BD Biosciences Cat#564796, RRID: AB_2738956
Mouse anti-human Aiolos-PE, clone S50-895 BD Biosciences Cat# 564811, RRID:AB_2738966
Mouse anti-human Aiolos-PE-CF594, clone S50-895 BD Biosciences Cat# 567871, RRID:AB_2916774
Mouse anti-human Eomes-eFluor 660, clone WD1928 eBiosciences Cat# 50-4877-42, RRID:AB_2574229
Mouse anti-human Eomes-Pe-Cy5.5, clone WD1928 Invitrogen Cat# 35-4877-42, RRID:AB_2848321
Mouse anti-human Eomes-Pe-Cy7, clone WD1928 eBioscience Cat# 25-4877-42, RRID:AB_2573456
FcRy-AF700, rabbit-anti-human EMD Milipore Mili-Mark Conjugated in house
Mouse anti-human Granzyme B-Pe-CF594, clone GB11 BD Biosciences Cat#561142, RRID: AB_10561690
Mouse anti-human Granzyme B-Pe-TexasRed, clone GB11 Invitrogen Cat# GRB17, RRID:AB_1500187
Mouse anti-human Granzyme B-BB790 BD Biosciences Custom Conjugate, Filipovic et al. (2019)44
Mouse anti-human HLA-A2-FITC, clone BB7.2 BD Biosciences Cat#343304, RRID: AB_1659245
Recombinant anti-human HLA-A2-Biotin, clone REA517 Miltenyi Biotec Cat#130-123-242, RRID: AB_2811481
Recombinant anti-human HLA-A3-FITC, clone REA950 Miltenyi Biotec Cat#130-115-793, RRID: AB_2727190
Mouse anti-human HLA-A25/26(A10)-Biotin OneLambda Cat# BIH0048
Mouse anti-human HLA-A11-Biotin OneLambda Cat#BIH0084
Mouse anti-human HLA-B7-FITC, clone BB7.1 Invitrogen Cat# MA1-82180, RRID:AB_931644
Recombinant anti-human HLA-B8-Biotin, clone REA145 Miltenyi Biotec Cat#130-099-589, RRID: AB_2652007
Recombinant anti-human HLA-B8-FITC, clone REA145 Miltenyi Biotec Cat#130-118-366, RRID: AB_2733668
Mouse anti-human HLA-B12-FITC OneLambda Cat#FH0066
Recombinant anti-human HLA-B12-Biotin Miltenyi Biotech Cat#130-099-856, RRID: AB_2652110
KIR2DL1-APC-Vio770, clone REA284 Miltenyi Biotech Cat#130-118-345, RRID: AB_2751487
Mouse anti-human KIR2DL1/S1-PC5.5, clone EB6B Beckman Coulter Cat# A66898, RRID:AB_2857330
Mouse anti-human KIR2DL2/L3/S2-PC5.5, clone GL183 Beckman Coulter Cat# A66900, RRID:AB_2857331
KIR3DL1- PerCP-Cy5.5, clone DX9 BD Pharmingen N/A (discontinued product)
Mouse anti-human KIR3DL1/S1-PE, clone Z27.3.7 Beckman Coulter Cat# IM3292, RRID:AB_131339
Mouse anti-human Ki-67-AF700, clone B56 BD Biosciences Cat#561277, RRID: AB_10611571
Mouse anti-human Ki-67-BV480, clone B56 BD Biosciences Cat# 566109, RRID:AB_2739511
Mouse anti-human Ki-67-RB780, clone B56 BD Biosciences Cat# 568761, RRID:AB_3684525
Mouse anti-human KLRG1-BV605, clone 2F1 Biolegend Cat# 138419, RRID:AB_2563357
Mouse anti-human NKG2A-AF488, clone 131411 R&D Systems Cat# FAB1059G-100
Mouse anti-human NKG2A-AF700, clone 131411 R&D Systems Cat# FAB1059N-100
Mouse anti-human NKG2A-APC, clone Z199 Beckman Coulter Cat# A60797, RRID:AB_10643105
Recombinant anti-human NKG2A- VioBright FITC, clone REA110 Miltenyi Biotec Cat#130-113-568, RRID: AB_2726173
Recombinant anti-human NKG2A-PE, clone REA110 Miltenyi Biotec Cat#130-113-566, RRID: AB_2726171
Mouse anti-human NKG2A-PE, clone Z199 Beckman Coulter Cat# IM3291U, RRID:AB_10643228
Mouse anti-human NKG2A-Pe-Cy7, clone Z199 Beckman Coulter Cat# B10246, RRID:AB_2687887
Mouse anti-human NKG2A-BB700, clone 131411 BD Biosciences Cat#747926, RRID: AB_2872387
Recombinant anti-human NKG2C- VioBright FITC, clone REA205 Miltenyi Biotec Cat#130-117-707, RRID: AB_2728023
Mouse anti-human NKG2D-PE-Cy7, clone 1D11 Biolegend Cat#320812, RRID: AB_2234394
Mouse anti-human NKp30-BV605, clone p30-15 BD Biosciences Cat#563384, RRID: AB_2738170
Mouse anti-human NKp44-BUV737, clone p44-8 BD Biosciences Cat# 744301, RRID:AB_2742131
Mouse anti-human NKp44-BUV737, clone p44-8 BD Biosciences Cat# 749172, RRID:AB_2873552
Mouse anti-human NKp46-BV786, clone 9E2/NKp46 BD Biosciences Cat#563329, RRID: AB_2738139
Mouse anti-human NKp46-BV650, clone 9E2 Biolegend Cat# 331927, RRID:AB_2562442
Mouse anti-human NKp80-APC, clone 5D12 Biolegend Cat#346708, RRID: AB_2044041
Mouse anti-human PD-1-BV421, clone EH12.2H7 Biolegend Cat#329920, RRID: AB_10960742
Mouse anti-human PD-1-BV785, clone EH12.2H7 Biolegend Cat# 329930, RRID:AB_2563443
Mouse anti-human Perforin-BV421, clone dG9 Biolegend Cat#308122, RRID: AB_2566204
Mouse anti-human Perforin-BV421, clone B-D48 Biolegegend Cat# 353307, RRID:AB_11149688
Mouse anti-human Perforin-PE-Cy7, clone B-D48 Biolegend Cat#353316, RRID: AB_2571973

Mouse anti-human Perforin-BB755, clone deltaG9 BD Biosciences Custom Conjugate, Filipovic et al. (2019)44
Mouse anti-human Runx3-PE, clone R3-5G4 BD Biosciences Cat# 564814, RRID:AB_2738969
Mouse anti-human Sialyl Lewis X-BV711, clone CSLEX1 BD Biosciences Cat#563910, RRID: AB_2738481
Recombinant anti-human Siglec-7-PercP-Vio 700, clone REA214 Miltenyi Biotec Cat#130-100-979, RRID: AB_2657543
Mouse anti-human Syk-PE, clone 4D10.1 Affymetrix eBiosciences (now Life Technologies) Cat#15557076
Mouse anti-human T-bet-PE-Dazzle 594, clone 4B10 Biolegend Cat#644828, RRID: AB_2565677
Mouse anti-human T-bet-BV421, clone 4B10 Biolegend Cat# 644815, RRID:AB_10896427
Mouse anti-human TIGIT-PE-Cy7, clone 1G9 Biolegend Cat#142108, RRID: AB_2565648
Mouse anti-human Tim-3-BV650, clone F38-2E2 Biolegend Cat#345028, RRID: AB_2565829
Mouse anti-human TCR PAN γ/d-PC5.5, clone IMMU510 Beckman Coulter Cat# A99021, RRID:AB_2910257
Mouse anti-human TCRVa7.2-PE-Cy7, clone 3C10 Biolegend Cat#351712, RRID: AB_2561994
Rat anti-mouse CD45-AF700, clone 30F11 Biolegend Cat#103128, RRID: AB_493715
Mouse anti-human/monkey CD3-AP-Cy7, clone SP34-2 BD Biosciences Cat#557757, RRID: AB_396863
Mouse anti-human/monkey CD3-BUV395, clone SP34-2 BD Biosciences Cat# 564117, RRID:AB_2738603
Mouse anti-human CD3-BV570, clone UCHT1 Biolegend Cat# 300436, RRID:AB_2562124
Mouse anti-human/monkey CD8a-BV570, clone RPA-T8 Biolegend Cat#301038, RRID: AB_2563213
Mouse anti-human/monkey CD8a-BUV496, clone RPA-T8 BD Biosciences Cat# 612942, RRID:AB_2870223
Mouse anti-human/monkey CD14-APC-Cy7, clone M5E2 Biolegend Cat#301820, RRID: AB_493695
Mouse anti-human/monkey CD14-BV510, clone M5E2 Biolegend Cat# 301842, RRID:AB_2561946
Mouse anti-human/monkey CD16-BV650, clone 3G8 Biolegend Cat#302042, RRID: AB_2563801
Mouse anti-human/monkey CD16-BUV496, clone 3G8 BD Biosciences Cat# 564653, RRID:AB_2744294
Mouse anti-human/monkey CD20-APC-Cy7, clone 2H7 Biolegend Cat#302314, RRID: AB_314262
Mouse anti-human/monkey CD20-PerCP/Cy5.5, clone 2H7 Biolegend Cat# 302326, RRID:AB_893283
Mouse anti-human/monkey CD56-PE-Cy7, clone B159 BD Biosciences Cat#557747, RRID: AB_396853
Mouse anti-human Granzyme B-PE Texas Red, clone GB11 Invitrogen Cat# GRB17, RRID:AB_2536540
Mouse anti-human Granzyme B-AF647, clone GB11 BD Biosciences Cat# 560212, RRID:AB_11154033
Mouse anti-human/monkey HLA-DR-BV421, clone G46-6 BD Biosciences Cat#562804, RRID: AB_2687421
Mouse anti-human/monkey HLA-DR-BV605, clone G46-6 BD Biosciences Cat#562845
Mouse anti-human/monkey HLA-DR-BV650, clone L243 Biolegend Cat# 307650, RRID:AB_2563828
Mouse anti-human/monkey HLA-DR-BUV615, clone G46-6 BD Biosciences Cat# 751142, RRID:AB_2875168
Mouse anti-human/non-human primates Perforin-FITC, clone Pf-344 Mabtech AB Cat#3465-7
Streptavidin-BB630 BD Biosciences Costum Conjugate, Filipovic et al. (2019)44
Streptavidin-QD585 Life Technologies Cat#Q10111MP

Validation

All antibodies are commercially available and are validated by the respective vendor on their official website.
BD Biosciences: https://www.bdbiosciences.com/en-eu/products/reagents/flow-cytometry-reagents/research-reagents/quality-and-reproducibility
Biolegend: https://www.biolegend.com/en-us/quality/quality-control
Miltenyi Biotec: https://www.miltenyibiotec.com/SE-en/products/macs-antibodies/antibody-validation.html
Beckman Coulter: https://www.beckman.com/reagents/coulter-flow-cytometry/antibodies-and-kits/single-color-antibodies/quality-standards
ThermoFisher Scientific: https://www.thermofisher.com/se/en/home/life-science/antibodies/invitrogen-antibody-validation.html
Merck Milli Pore: https://www.merckmillipore.com/SE/en/life-science-research/antibodies-assays/antibodies-overview/Antibody-Development-and-Validation/cFOb.qB.8McAAAFOb64qQvSS,nav

Animals and other research organisms

Policy information about studies involving animals; ARRIVE guidelines recommended for reporting animal research, and Sex and Gender in Research

Laboratory animals

MISTRG mice homozygous for the human genes encoding M-CSF, IL-3, GM-CSF, SIRPα, and TPO in the Rag2−/−Il2rg−/− background were previously described (Rongvaux et al., 2014). MISTRG mice were used under Material Transfer Agreements with Regeneron Pharmaceuticals and Yale University.
Rhesus macaques of Indian origin were obtained from the University of Pennsylvania.

Wild animals

This study did not involve wild animals.

Reporting on sex

Information not collected.

Field-collected samples

This study did not involve field-collected samples.

Ethics oversight

All mouse experiments were approved by the Linköping Animal Experimentation Ethics Committee and performed in accordance with local guidelines.
All macaque studies were approved by the Institutional Animal Care and Use Committee at the University of Pennsylvania and all procedures were conducted in accordance with the Animal Welfare Act and other US federal statutes and regulations relating to animals. Animal care facilities at Emory National Primate Research Center are accredited by the U.S. Department of Agriculture (USDA) and the Association for Assessment and Accreditation of Laboratory Animal Care (AAALAC) International.

Note that full information on the approval of the study protocol must also be provided in the manuscript.

Plants

Seed stocks

Report on the source of all seed stocks or other plant material used. If applicable, state the seed stock centre and catalogue number. If plant specimens were collected from the field, describe the collection location, date and sampling procedures.

Novel plant genotypes

Describe the methods by which all novel plant genotypes were produced. This includes those generated by transgenic approaches, gene editing, chemical/radiation-based mutagenesis and hybridization. For transgenic lines, describe the transformation method, the number of independent lines analyzed and the generation upon which experiments were performed. For gene-edited lines, describe the editor used, the endogenous sequence targeted for editing, the targeting guide RNA sequence (if applicable) and how the editor was applied.

Authentication

Describe any authentication procedures for each seed stock used or novel genotype generated. Describe any experiments used to assess the effect of a mutation and, where applicable, how potential secondary effects (e.g. second site T-DNA insertions, mosiacism, off-target gene editing) were examined.

Flow Cytometry

Plots

Confirm that:

☒ The axis labels state the marker and fluorochrome used (e.g. CD4-FITC).

☒ The axis scales are clearly visible. Include numbers along axes only for bottom left plot of group (a 'group' is an analysis of identical markers).

☒ All plots are contour plots with outliers or pseudocolor plots.

☒ A numerical value for number of cells or percentage (with statistics) is provided.

Methodology

Sample preparation

Peripheral blood mononuclear cells (PBMCs) were isolated from PB and efferent lymph fluid using Ficoll Hypaque gradient centrifugation and washed in PBS.
Duodenum, uterine, liver-draining lymph nodes and liver tissues were cut into small pieces, transferred into complete RPMI 1640 medium and enzymatically digested using Collagenase II and DNAse. Tonsils were cut and mashed through a 100 um nylon cell strainer into complete RPMI medium. All tissue-derived cell suspensions were additionally filtered through a 70 um cell strainer.
Cells were subsequently stained with surface antibodies and fixed. If ICS was carried out cells were permeabilized and incubated with intracellular antibodies.

Instrument

Stained samples were measured on a BD LSR Fortessa, BD LSRII, BD FACSymphony A5 (all BD Biosciences) or Navios flow cytometer (Beckman Coulter). NK cell populations were sorted on a BD Aria (BD Biosciences) at the MedH FACS core facility, Karolinska Institutet.

Software

Standard flow cytometry data analysis was performed using FlowJo version 10.4.2. (BD Biosciences) or Kaluza software 1.5a (Beckman Coulter, Brea, CA, USA). Analysis and visualization of surface marker expression on NK cell subsets was further performed in R Studio (v1.3.959) using the packages ggplot2 (v3.3.2), reshape2 (v1.4.4), tidyr (v1.1.1), viridis (v0.5.1) and dplyr (v1.0.1).

Cell population abundance

Human CD56bright and CD56dim NK cells were sorted on a an Aria Fusion (BD Biosciences) and subsequently underwent TGF-beta stimulation. For CRISPR/Cas9 treatment, human peripheral blood NK cells were enriched using the human NK cell isolation kit (Miltenyi Biotec).

Gating strategy

Exemplary gating strategies are provided in Supplementary Figure 1 and 2.

Samples were gated on time (Time vs. SSC-A) for data cleaning. Cells populations were gated using FSC-A vs. SSC-A and single cells were identified using FSC-A vs. FSC-H or SSC-A vs. SSC-H gating. Dead cells were excluded and lymphocytes were identified via CD45 (DCM vs. CD45).

Subsequent gating strategies were dependent on the individual cohort and experiment strategy and illustrated in the exemplary gating strategies, highlighted in representative plots in Figure 2, 3 and 6 or are described in the manuscript.

☒ Tick this box to confirm that a figure exemplifying the gating strategy is provided in the Supplementary Information.
